# Non-Hermitian singularities in scattering spectra of Mie resonators

**DOI:** 10.1126/sciadv.adr9183

**Published:** 2025-02-21

**Authors:** Fan Zhang, Nikolay S. Solodovchenko, Hangkai Fan, Mikhail F. Limonov, Mingzhao Song, Yuri S. Kivshar, Andrey A. Bogdanov

**Affiliations:** ^1^ Qingdao Innovation and Development Center, Harbin Engineering University, Qingdao 266000, Shandong, China.; ^2^ School of Physics and Engineering, ITMO University, St. Petersburg 191002, Russia.; ^3^ Ioffe Institute, St. Petersburg 194021, Russia.; ^4^ Nonlinear Physics Center, Australian National University, Canberra, ACT 2601, Australia.

## Abstract

Non-Hermitian systems are known to have unique singularities, notably exceptional points. Mie resonators demonstrate fruitful electromagnetic multipole interference effects in scattering behavior. The research of these non-Hermitian singularities is typically conducted independently with the analysis of scattering interference. Here, we demonstrate fundamental relationships between non-Hermitian singularities and observe their manifestation in the scattering spectra. We reveal that exceptional points always exist in the anapole regime, and diabolic points are associated with superscattering. We confirm our theoretical findings in the microwave experiment by measuring the extinction spectra of subwavelength Mie-resonant ceramic rings. Our study underpins the generic behavior of non-Hermitian singularities in the scattering spectra of subwavelength Mie resonators, uncovering their special applications in non-Hermitian nonlinear optics and topological photonics.

## Teaser

Exploring the non-Hermitian singularities in the scattering spectra of Mie resonators reveals new paths for field manipulation.

## INTRODUCTION

The study of Mie resonances in subwavelength dielectric particles has substantially advanced our understanding of the scattering and localization of electromagnetic waves (*1*, *2*). In particular, the subwavelength resonators can support multipole electric and magnetic Mie resonances, and their control and engineering allow manipulating light-matter interaction at a subwavelength scale (*3*–*5*). Each Mie resonator is an open physical system that, due to radiative losses, is characterized by complex eigenvalues, and thus it becomes an object of non-Hermitian physics (*6*). Complex eigenvalues can be manipulated through the meticulous tailoring of the structural parameters, thereby enabling a variety of exotic phenomena driven by coupling and interference between multipolar resonances, including quasi-bound states in the continuum (Q-BICs), which theoretically have ultrahigh quality factors and the near-field localization (*7*–*9*), the unidirectional scattering Kerker effect (*10*, *11*), anapole states characterized by the scattering dark state (*12*), superscattering (*13*), and supermultipole (*14*), which can break the limit of single-channel scattering.

Non-Hermitian physics is known to offer many distinctive phenomena that are not accessible in Hermitian systems (*15*). In particular, exceptional points (EPs) are unique singularities in non-Hermitian systems where the eigenvalues and eigenvectors coalesce (*16*–*19*). This contrasts with more typical spectral accidental degeneracies, such as diabolic points (DPs) in Hermitian systems, where only the real eigenvalues are degenerate, whereas the eigenstates remain linearly independent (*20*). These singularities give rise to various novel physical phenomena, such as chiral mode switching (*21*–*23*), unidirectionality (*24*–*26*), nonreciprocal wave propagation (*27*), coherent perfect absorption (*28*–*30*), and enhanced sensitivity (*31*, *32*), making them a focal point in numerous disciplines, including acoustics (*33*), mechanics (*34*), electronics (*35*), semiconductor physics (*36*, *37*), photonics (*38*–*40*), and plasmonics (*41*–*45*). EPs are closely associated with the concept of parity-time (PT) symmetry, providing the critical link between symmetric and broken PT phases. Although traditionally associated with gain-loss balance, it can also be realized in passive PT-symmetric systems (*46*, *47*). In particular, EPs originating from Mie resonances have been studied only theoretically (*48*, *49*), and these studies are limited by the analysis of eigenvalues and evolution of the scattering spectrum. The unique features of non-Hermitian wave scattering from Mie resonators were never demonstrated in the experiment.

From the perspective of multipole channel coupling, it is essential to elucidate different phenomena associated with Mie resonances and non-Hermitian singularities. Radiative coupling can be decomposed into multipolar radiation, and by examining the non-Hermitian systems from a multipole perspective, we can gain an understanding of the effect of complex eigenmodes. Here, we numerically and experimentally study non-Hermitian Mie singularities. By an example of a dielectric ring–shaped resonator, we explore the transition between EPs and DPs by manipulating structural parameters to control the system detuning and mode coupling and to observe and use EPs. The primary objective of this study is to elucidate the dynamics of non-Hermitian singularities in dielectric ring resonators and to demonstrate their potential applications in controlling and enhancing light-matter interactions. By understanding the underlying mechanisms and characteristics of EPs and DPs, we aim to pave the way toward the development of highly tunable sensors and other optical devices that leverage these unique non-Hermitian properties. We believe our results not only contribute to the fundamental understanding of the insight physics of non-Hermitian singularities in Mie scattering but also expand opportunities for potential applications in various optical technologies. The ability to manipulate and use the scattering behavior of non-Hermitian singularities in dielectric resonators promises to revolutionize the design and functionality of next-generation photonic devices.

## RESULTS

**Fig. 1.**
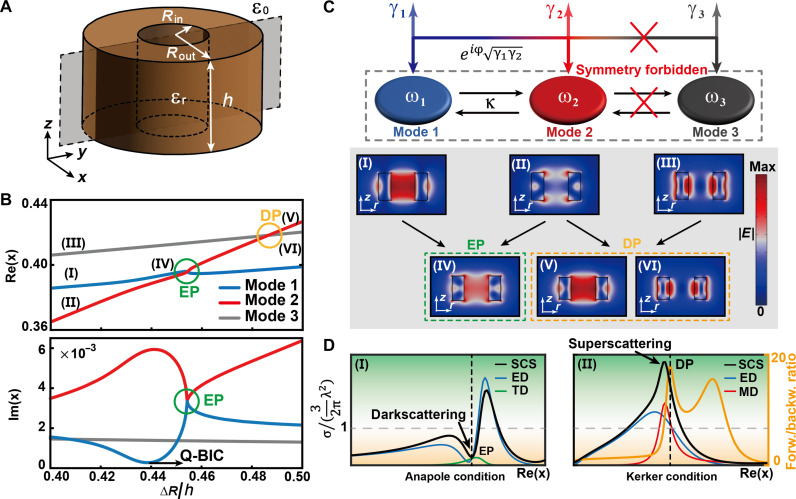
Non-Hermitian singularities in a single ring-shaped dielectric resonator. (**A**) Schematic illustration of the proposed ring-shaped dielectric resonator. (**B**) Simulated complex size parameter x=ω∆R/c=2πf∆R/c of three eigenmodes as the functions of varying aspect ratio ∆R/h. EP can be predicted at the position where both real and imaginary parts of x coalesce (∆R/h = 0.4524), whereas DP appears when only the real part of *x* merge (∆*R*/*h* = 0.4865). (**C**) General concept of the TCMT describing the proposed non-Hermitian system, which consists of the closed system circled by the black dashed line and the radiative losses. The electric-field distributions along the cross section in the gray plane of (A) correspond to the eigenmodes of (B), where eigenstates coalesce at EP. (**D**) Scattering behavior of the anapole state at EP and the superscattering state at DP.

As an example, we consider the structure illustrated in Fig. 1A in the form of a dielectric ring–shaped subwavelength Mie resonator with permittivity (ε_*r*_>) of 80 placed in the air (ε_0_ = 1), which brings a realization of a non-Hermitian system. For theoretical investigation of the singularities in such a non-Hermitian system, we will use a standard effective Hamiltonian with three resonances accounting for the near- and far-field coupling between the modes (Fig. 1C). The model assumes the existence of three distinct modes, the first two of which exhibit the same symmetry and thus near-field and far-field coupling. The last mode, however, exhibits a different symmetry, which precludes any interaction due to symmetry prohibitions, ensuring the orthogonality between these modes. The theoretical framework used to describe this system is the temporal coupled-mode theory (TCMT), and the dynamics of the open system can be described using a Hamiltonian matrix

$$\hat{H}=\left(\begin{array}{ccc} \omega_{1} & \kappa & 0\\ \kappa & \omega_{2} & 0\\ 0 & 0 & \omega_{3} \end{array}\right)-i\;\left(\begin{array}{ccc} \gamma_{1} & e^{i\psi }\sqrt{\gamma_{1}\gamma_{2}} & 0\\ e^{i\psi }\sqrt{\gamma_{1}\gamma_{2}} & \gamma_{2} & 0\\ 0 & 0 & \gamma_{3} \end{array}\right)$$ (1)

where the first term is the Hermitian part describing a lossless system, and the second term is the anti-Hermitian part describing radiation of two resonances with the same symmetry. ω_*j*_ and γ_*j*_ (*j* = 1,2,3) represent the resonant frequencies and leakage rates of the resonances. Parameter κ denotes the near-field coupling strength between two symmetric modes, $e^{i\psi }\sqrt{\gamma_{1}\gamma_{2}}$ is the interference of the radiative waves through far-field coupling, and $\sqrt{\gamma_{1}\gamma_{2}}$ is the radiative coupling term, whereas ψ is the phase difference between two symmetric modes. The complex eigenvalues of the Hamiltonian (Eq. 1) are

$$\left\{ \begin{array}{c} {\tilde{\Omega }}_{\pm }=\omega_{\text{ave}}-i\gamma_{\text{ave}}\pm \sqrt{{\left(∆\omega -i∆\gamma \right)}^{2}+{\left(\kappa -e^{i\psi }\sqrt{\gamma_{1}\gamma_{2}}\right)}^{2}}\\ {\tilde{\Omega }}_{3}=\omega_{3}-i\gamma_{3} \end{array}\right.$$ (2)

where ${\tilde{\Omega }}_{\pm }$ represents the complex eigenvalues induced by the modes with same symmetry where the coupling is considered, and ${\tilde{\Omega }}_{3}$ represents the complex eigenvalue of the mode with opposite symmetry without coupling. $\omega_{\text{ave}}=\left(\omega_{1}+\omega_{2}\right)/2$ and $\gamma_{\text{ave}}=\left(\gamma_{1}+\gamma_{2}\right)/2$are the averages of $\omega_{j}$ and $\gamma_{j}$ (*j* = 1,2), whereas $\Delta \omega =\left(\omega_{1}-\omega_{2}\right)/2$ and $\Delta \gamma =\left(\gamma_{1}-\gamma_{2}\right)/2$ are the differences of $\omega_{j}$ and $\gamma_{j}$ (*j* = 1,2), respectively.

Learned from Eq. 2, the complex eigenvalues can be freely tailored by manipulating the detuning and coupling regardless of the structure. As an example of the proposed single dielectric ring resonator, one of the simplest ways to control the detuning and coupling is varying the structural parameters [i.e., aspect ratio $\left(R_{\text{out}}-R_{\text{in}}\right)/h=∆R/h$ and radius ratio $R_{\text{in}}/R_{\text{out}}$]. The external radius $R_{\text{out}}$ is fixed to guarantee that only two degrees of freedom in the proposed system. Figure 1B shows the critical coupling strength ($R_{\text{in}}/R_{\text{out}}$ = 0.4983) by changing ∆R/h; EP appears at the critical state characterized by the coalescing of both real and imaginary parts of the eigenvalues indicated by the green circles [Fig. 1B(IV)]. DP appears at the crossing point where only the real eigenvalues merge, indicated by the yellow circle [Fig. 1B(VI)]. At ∆*R*/*h* = 0.4524, the electric-field distributions of the two eigenmodes are the same [Figs. 1C(IV)] because of the coalescing of the eigenvectors at EP, whereas at ∆*R*/*h* = 0.4865, the electric-field distributions of the two eigenmodes are different because the associated eigenvectors are orthogonal [Figs. 1C(V) and 1C(VI)]. In Fig. 1D, we explain the scattering behavior at EP and DP associated with multipole Mie resonances. EP is related to the dark scattering state that meets the anapole condition, whereas DP is related to the super scattering state that meets the Kerker condition.

**Fig. 2.**
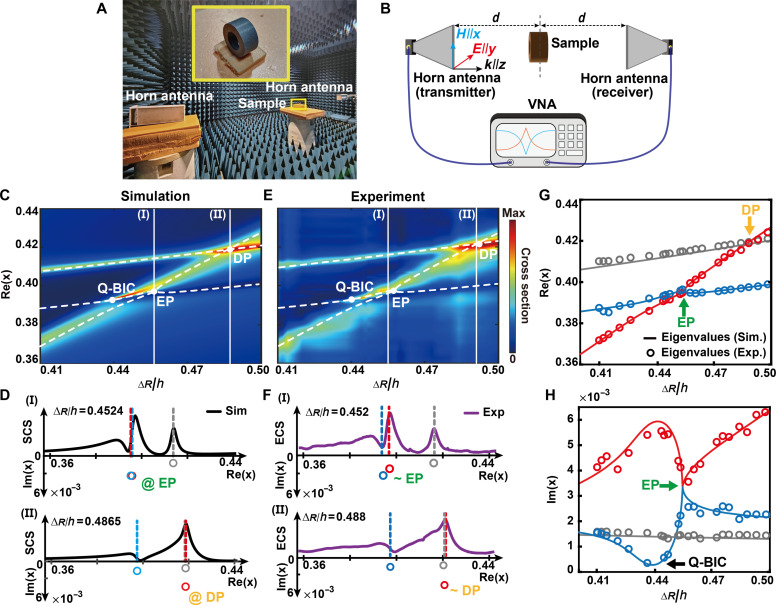
Far-field numerical and experimental results. (**A**) Far-field experimental setup and (**B**) schematic illustration. (**C**) Simulated map of the ECS under the normal incident wave with the dependency of ∆R/h and x. The white dashed lines are visual auxiliary lines of the eigenvalues. The white dots indicate the position of EP and DP. (**D**) Panels of the simulated ECS at EP and DP. (**E**) Experimental map of the scattering cross section under the normal incident wave with the dependency of ∆R/h and x. (**F**) Panels of the experimental ECS near EP and DP. (**G** and **H**) Real (G) and imaginary (H) parts of the simulated and experimental eigenvalues. The colorful solid lines represent the simulated eigenvalues, whereas colorful dots represent extracted eigenvalues from experimental results.

To demonstrate the existence of the EP in above system and characterize the scattering behavior, we consider plotting the scattering cross-sectional maps of a lossless dielectric ring–shaped resonator with varying ∆*R*/*h* in simulation, shown in Fig. 2C. The white dashed lines indicate three types of the simulated eigenvalues, which are the same with Fig. 1B. Two crossing points (i.e., EP and DP) can be found but locate at the dip and peak of the extinction cross section (ECS), respectively. Figure 2D shows the induvial ECS curves at EP [along Fig. 2C(I)] and DP [along Fig. 2C(II)]. At ∆*R*/*h* = 0.4524, EP is located on the dip in the ECS between two resonance peaks, a phenomenon that is similar to coupled-resonator-induced transparency and electromagnetically induced transparency. At EP, two complex eigenvalues (blue and red) coalesce in the eigenvalue space. At ∆*R*/*h* = 0.4865, DP is situated at the overlapping of resonance peaks, which is analogous to the superscattering state. At DP, only the real part of two complex eigenvalues (gray and red) merge.

For the experimental verification, we fabricate a set of dielectric ring–shaped samples (the external radius $R_{\text{out}}$ = 1.6 cm, *R*_in_/*R*_out_ = 0.498) with a varying height; the selected compound material is $\text{BaO}-{\text{Ln}}_{2}O_{3}-\text{Ti}O_{3}$ with a permittivity ε = 80.05 and loss tangent δ = 1.8 × 10^−4^. Details about the sample fabrication and far-field experimental setup can be found in Materials and Methods. The measured ECS for the normal incident wave is plotted in Fig. 2E and shows good agreement with the numerical simulation results in the top panel, with differences due to the scattering from the inhomogeneity, fabrication defect, and the uncertainty of the measurement environment. Experimental fitting results (white solid lines of Fig. 2E) show a tiny displacement of the two crossing points of Re(${\tilde{\Omega }}_{\pm }$), at approximately ∆*R*/*h* = 0.452 and ∆*R*/*h* = 0.488, due to the precision limits of sample preparation. In Fig. 2F, we investigate the evolution of ECS results from the experiment (purple solid lines) with the corresponding complex eigenvalues at different ∆R/h. At ∆*R*/*h* = 0.452, two complex eigenvalues (blue and red) are close, indicating the presence near the EP. At ∆*R*/*h* = 0.488, two complex eigenvalues (gray and red) degenerate only in the real part, representing close to DP.

To more accurately obtain more information from experimental curves, we consider a method based on quasi-normal modes (QNMs) (*50*), which allows us to obtain the extinction spectrum from each eigenmode separately. The response from each QNM is fitted by using the Fano formula, and all the necessary resonance parameters are obtained: Fano parameter q, intensity *I*, and eigenfrequency $\omega_{j}-\gamma_{j}$ (*j* = 1,2,3). The data obtained are used as an initial approximation of parameters for the purpose of fitting experimental spectra, with the objective of obtaining frequencies and radiation losses. Figure 2 (G and H) compares the simulation and experimental normalized complex eigenvalues with different ∆R/h (with detailed comparison between fitted and measured ECS curves in the Supplementary Materials). The excellent agreement shows the validity of this QNM method. The difference between the numerically calculated complex eigenvalues and the experimental results mainly stems from the scattering from the sample surface roughness and inhomogeneity in fabrication.

**Fig. 3.**
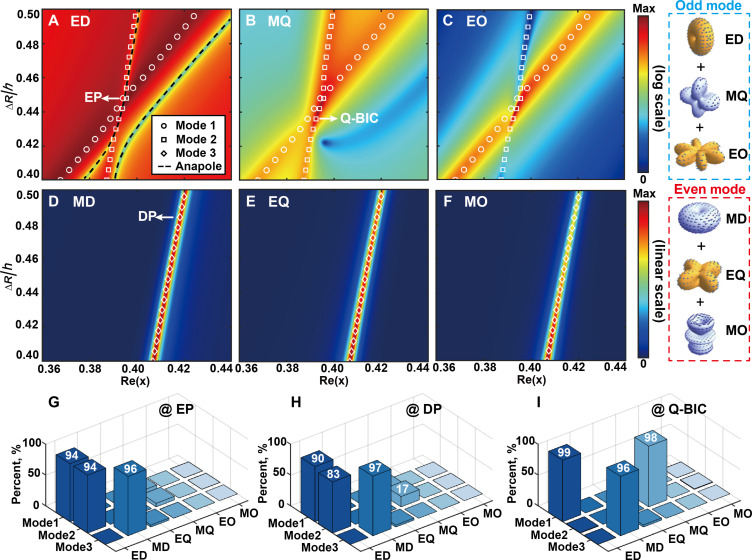
Multipole analysis. The ED (**A**), magnetic quadrupole (MQ) (**B**), electric octupole (EO) (**C**), MD (**D**), electric quadrupole (EQ) (**E**), and magnetic octupole (MO) (**F**) contribution of the scattering cross section excited by the normal incident wave with the dependency of aspect ratio ∆R/h and size parameter x. The various types of white dots indicate three simulated eigenmodes. The black dashed lines indicate the position of scattering dark states in the ED radiation channel. The right panels show that multipoles can be divided into odd (blue) and even (red) multipolar in an inversion symmetric particle. (**G** to **I**) Proportion of multipolar at different singularities: (G) EP, (H) DP, and (I) Q-BIC.

Learning from the behavior of scattering spectra, we understand that EP appears in the dip between two resonance peaks [Fig. 2D(I)/Fig. 2F(I)], whereas DP appears in the overlap of two resonance peaks [Fig. 2D(II)/Fig. 2F(II)]. To gain a deeper insight into the scattering behavior of these singularities, we have used multipole decomposition. Figure 3 (A to F) illustrates the first three orders of multipoles for the ECS of the simulation in Fig. 2C. The ring-shaped dielectric resonator exhibits inversion symmetry, which results in the eigenmodes of the resonator comprising either only odd (blue dashed frame in the right panels of Fig. 3) or even (red dashed frame in the right panels of Fig. 3) multipoles (*51*, *52*). Figure 3 (A to C) illustrates the primary multipoles of odd modes. Two odd modes (white circles and squares), which are predominantly attributed to the electric dipole (ED), are observed to degenerate at the position of EP. Figure 3G illustrates the proportion of multipolars at EP of three modes. The destructive interference between two EDs contributed odd modes (mode 1 and mode 2) in the far-field results in the scattering dark state (i.e., anapole state) in ECS between the positions of two eigenmodes, as illustrated in Fig. 3A by the black solid lines. It is also worth mentioning that Q-BIC appears during the multipolar conversion from dipole to quadrupole (mode 2) shown in Fig. 3I. A comparable phenomenon is also observed in two magnetic dipoles (MDs), as shown in the Supplementary Materials. Figure 3 (D to F) shows the main multipoles of even modes (white diamonds). The DP is observed at the crossing position where an odd mode (mode 1) merges with an even mode (mode 3). This phenomenon can be described as the superposition of ED and MD, as shown in Fig. 3H. Because ED and MD are not subject to interference in the far field, the scattering behavior in the ECS can be considered as the superposition of resonance peaks. Further details on the quantification of multipoles at different singularities can be found in the Supplementary Materials.

**Fig. 4.**
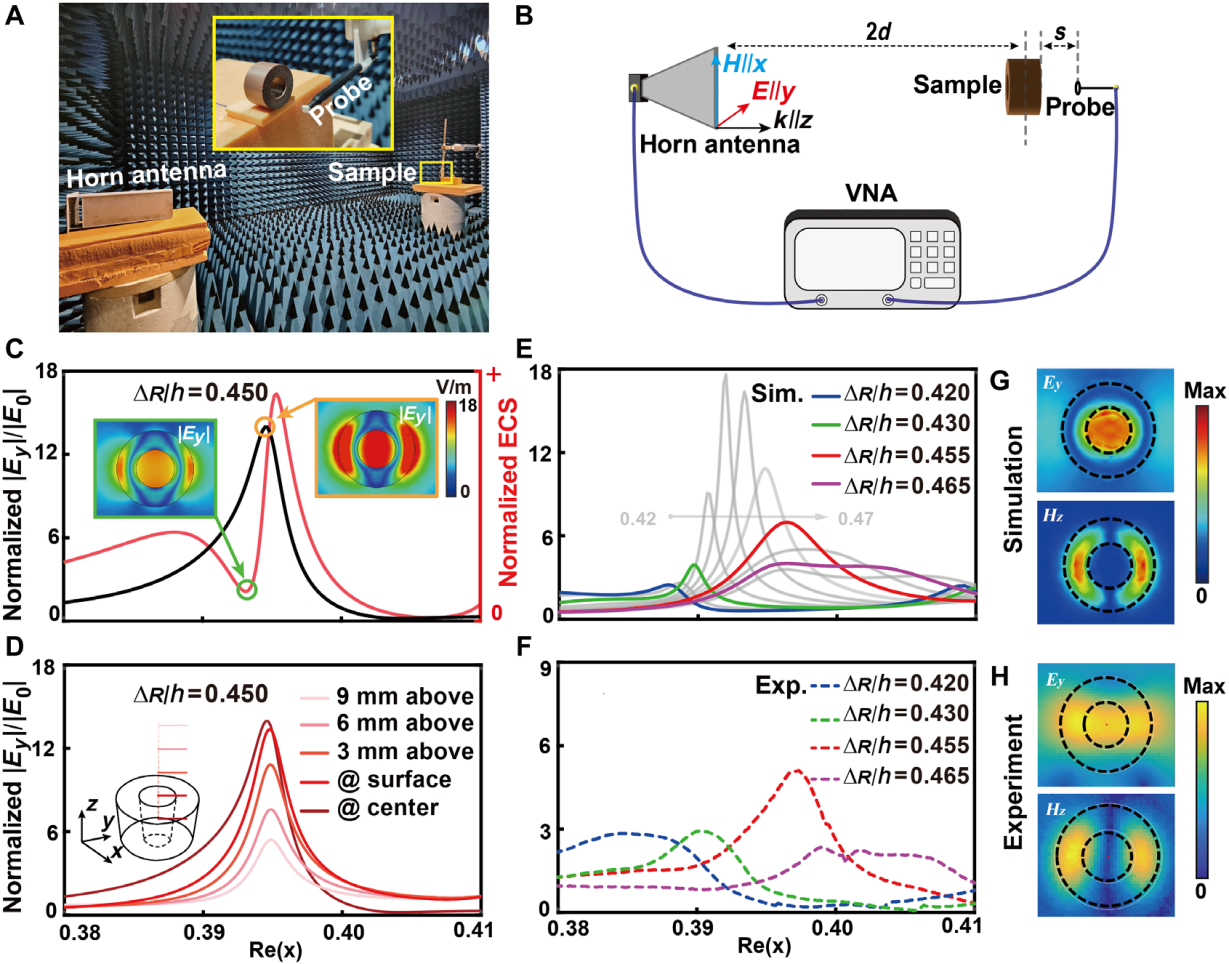
Near-field numerical and experimental results. (**A**) Near-field experimental setup and (**B**) schematic illustration. (**C**) Calculated normalized ECS and electric-field intensity enhancement in the center of the ring-shaped resonator. The insets show the *y* component of electric-field distribution at the dip of the scattering cross section and the peak of the electric-field enhancement. (**D**) Evolution of the *y* component of the electric-field enhancement with the observation points changed in height. The simulated (**E**) and the corresponding experimental (**F**) results of the evolution of $|E_{y}|/|E_{0}|$ with continuous changing aspect ratio. The simulated (**G**) and experimental (**H**) profiles of *y* component of electric field and *z* component of magnetic field 3 mm above the top surface of the resonator.

The location of far-field scattering suppression tends to result in a pronounced near-field enhancement effect (especially anapole state), and therefore, we consider conducting near-field detection experiments on the proposed ring resonator. Details about the near-field experimental setup and sketch can be found in Fig. 4 (A and B) and Materials and Methods. First, a numerical study is conducted on the normalized ECS (red line in Fig. 4C) and the *y* component of the near-electric-field enhancement in the center position inside the ring-shaped resonator (black line in Fig. 4C). It is observed that the position of the dip in the normalized ECS exhibits a slight difference to the maximum of the near-electric-field enhancement. Furthermore, the *y* component of the electric field exhibits a comparable profile, although with slightly different magnitudes. Consequently, the optimal position can be identified as that achieves the largest near-electric-field enhancement. In addition, we also investigate the evolution of field enhancement by varying the observation plane height to choose the best height for the experiment. As illustrated in Fig. 4D, a height of ~3 mm proved to be an optimal range for achieving field enhancement experimentally, with minimal attenuation on near-electric-field enhancement. In Fig. 4E, the $R_{\text{in}}/R_{\text{out}}$ of the sample is fixed at 0.4985, and the ∆*R*/*h* is uniformly changed from 0.42 to 0.47. This allows for straightforward control of the near-electric-field strength enhancement, which provides an effective method for enhancing and controlling light-matter interference. The experimental results are presented in Fig. 4F, where the colorful dashed lines demonstrate a satisfactory correlation with the simulated results, as indicated by the corresponding colorful solid lines shown in Fig. 4E. At 3 mm above the surface of the ring resonator, we investigate the *y* component of electric field and *z* component of magnetic field in both the simulation (Fig. 4G) and the experiment (Fig. 4H). It can be observed that there is a pronounced electric-field enhancement in the central surface of the resonator.

**Fig. 5.**
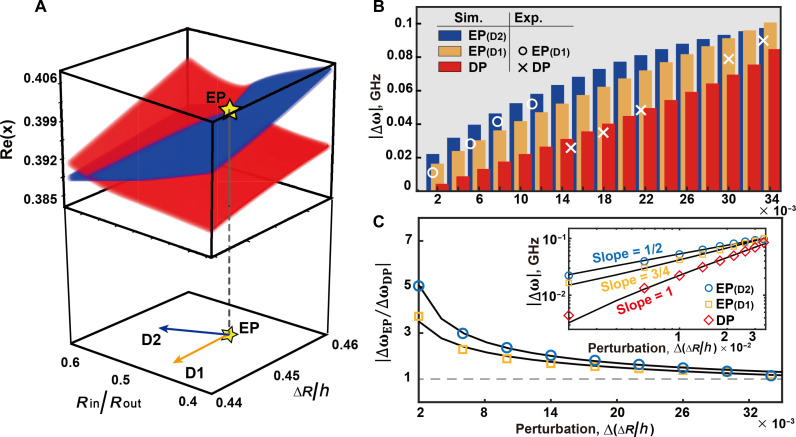
Robustness of the system sensitivity based on eigenfrequency splitting. (**A**) Evolution of the real part of the eigenvalues, where the golden star indicates the position of EP. The bottom panel indicates two directions (D1, direction of PT symmetry breaking; D2, direction when $R_{\text{in}}/R_{\text{out}}$ is fixed) of variation of the structural parameters around EP. (**B**) Histograms showing the eigenfrequency splitting in the ring-shaped resonator described by a comprehensive structural parameter at DP and EP sensors. The white circles and crosses represent the experimental data. (**C**) Dependence of the sensitivity enhancement $|∆\omega_{\text{EP}}/∆\omega_{\text{DP}}|$; the inset shows a double logarithmic plot between eigenfrequency splitting and perturbation at DP and EP, respectively.

We further investigate the sensitivity with varying structural parameters and discuss the system robustness of the sensitivity in the vicinity of EP. Figure 5A illustrates the evolution of Re(x) with varying ∆R/h and $R_{\text{in}}/R_{\text{out}}$, where the golden star indicates the position of EP. We consider two directions (D1 and D2) to demonstrate the robustness of the sensitivity at EP. D1 (yellow arrow) means fixed $R_{\text{in}}/R_{\text{out}}$ and varying ∆R/h, and D2 (blue arrow) means optimizing $R_{\text{in}}/R_{\text{out}}$ of every corresponding ∆R/h along the PT symmetry lines (in accordance with tables S1 and S2). Figure 5B demonstrates the simulated response to the perturbation, represented as the splitting degree of the eigenfrequency, of EP (blue and yellow) and DP (red) as histograms, whereas the scatterplots represent the experimental results. The red bar indicates the degree of response to changes in ∆R/h at DP. The red diamond in the inset in Fig. 5C indicates that the slope is 1, implying that the response to external perturbation is linear at DP sensing. When along the D1, the yellow bar displays the degree of resonance splitting at EP. The slope of yellow squares in the inset in Fig. 5C is 0.75, indicating that the limit of EP sensing has not been reached. When along the D2, the blue bar graph illustrates the degree of resonance splitting degree at EP when the PT symmetry condition is met. The blue circles in the inset in Fig. 5C, which exhibit a slope of 0.5, indicate a square root relationship. This implies that the maximum theoretically achievable nonlinear response at EP sensing has been reached. As illustrated in Fig. 5C, the maximum degree of response to perturbation at EP is more than five times higher than that at DP, according to theoretical calculations.

## DISCUSSION

We have uncovered the power of non-Hermitian singularities in the Mie scattering by studying the behavior of scattering around EPs and DPs through meticulous system detuning. By carefully actuating the height of a subwavelength dielectric ring, we have detected unique eigenvalue behaviors and field distributions and observed a distinct state transition between scattering dark states at EP and superscattering states at DP. In addition to our findings, we have proposed a general framework suggesting that the coalescence of modes with identical radiation patterns leads to the formation of EPs. Taking the example of ED-ED interactions, we have validated this hypothesis by demonstrating that EPs emerge at the ED anapole states. Furthermore, we have extended this concept to explore its applicability to modes involving magnetic or higher-order multipoles, highlighting its potential generality. Notably, we have experimentally verified and predicted the positions of EPs directly from extinction spectra, corroborating these predictions through QNM fitting. By bridging the concept of non-Hermitian singularities with multipole scattering interference, our work establishes a foundational connection within the emerging field of non-Hermitian Mie-tronics. Our study of non-Hermitian singularities highlights novel opportunities for developing advanced optical devices with enhanced sensitivity and control over light-matter interactions. Our results not only pave the way toward the development of compact and functional metadevices (such as sensors, absorbers, lasers, and energy harvesting/transmission units) with high sensitivity and tunable quality factors but also unveil unexplored avenues within the dynamic landscape of multipolar Mie resonances in non-Hermitian photonics (*53*).

## MATERIALS AND METHODS

### Numerical simulations

The full wave simulations to calculate both the eigenvalues and the ECS are performed in the Wave Optics module of COMSOL Multiphysics (commercial finite element simulation software). The dielectric resonator is surrounded by an air layer (more than one wavelength thick) and a 10-layer perfectly matched layer. A physically controlled mesh with extremely fine element size is used. Because the dielectric ring is inversion symmetric, we reduce the three-dimensional (3D) model to a 2D axisymmetric model, following the method introduced in (*54*). The electromagnetic fields can be expanded into a Fourier series of waves corresponding to different azimuthal indices. For the calculation of eigenvalues, the azimuthal number is set to 1. To calculate the ECS, the results are summed with the azimuthal number from −3 to 3, which can almost be considered as the total ECS.

### Description of the experiment details

The image of the sample is shown in the inset in Fig. 2A, placed on a foam plate holder, which can be considered transparent at a microwave frequency band. We perform the far-field experiment in an anechoic chamber over the microwave frequency band of 1.8 to 2.8 GHz. The photo and sketch of the experimental setup to measure the ECS of the ring-shaped resonator are shown in Fig. 2 (A and B). We choose a broadband horn antenna generating the normal incident plane wave as a transmitter to excite the sample and another antenna with the same configuration as a receiver, forming a two-port network. The distance d≈2 m, which is 10 times than the working wavelength, making the far-field condition. The sketch depicted in Fig. 2B illustrates the same experimental configuration of the experimental setup as shown in Fig. 2A. A vector network analyzer (VNA) is used to generate microwave and collect transmission spectra. Two horn antennas are served as a normal incident microwave transmitter and receiver respectively with linear polarization. The laser is used to collimate the horn antennas and measured sample. Before measuring transmission spectra, the VNA is calibrated without sample to subtract the background.

The ECS of the single dielectric resonator is measured in an anechoic chamber. The ECS was calculated from the measured complex transmission coefficient *S*_21_ (*55*, *56*)

$$\sigma_{\text{ext}}\left(f\right)=\frac{c}{\sqrt{\pi }f}\cdot \text{Im}\left(\frac{S_{21}}{S_{21,\text{bg}}}\right)$$ (3)

where σ_ext_ is the ECS, *c* is the light speed in the vacuum, *S*_21_ is the complex transmission coefficients of the resonator with background, and *S*_21,bg_ is the complex transmission coefficients of the background.

Figure 4 (A and B) depict the near-field experimental setup and sketch used to quantify the near-electric-field intensity. The ring-shaped samples are excited by a normal incident plane wave, and the probe for measuring the near-electric-field is positioned above the top surface of the sample. A VNA is used to generate microwave and collect *y* component of the near-electric-field distribution. One horn antenna is served as a normal incident microwave transmitter with linear polarization. The probe is *s* ≈ 3 mm, which is the depth of the probe, above the surface of the resonator.

## Supplementary Materials

### This PDF file includes:

Supplementary Text Figs. S1 to S10 Tables S1 and S2 References
