# Supplementary material for: Non-Hermitian singularities in scattering spectra of Mie resonators
Source: Sci Adv. 2025 Feb 21;11(8):eadr9183. doi: 10.1126/sciadv.adr9183 (PMC11844723; doi:10.1126/sciadv.adr9183)
Supplement: Supplementary file 1 — Supplementary Text Figs. S1 to S10 Tables S1 and S2 References [file sciadv.adr9183_sm.pdf]

Science Advances
AAAS

Supplementary Materials for

Non-Hermitian singularities in scattering spectra of Mie resonators

Fan Zhang *et al.*

Corresponding author: Mingzhao Song, kevinsmz@foxmail.com; Yuri S. Kivshar, yuri.kivshar@anu.edu.au; Andrey A. Bogdanov, bogdan.taurus@gmail.com

Sci. Adv. **11**, eadr9183 (2025)
DOI: 10.1126/sciadv.adr9183

This PDF file includes:

Supplementary Text
Figs. S1 to S10
Tables S1 and S2
References

Supplementary Text

S1. Eigenproblem of EPs in a two-mode coupled system with TCMT model

For the generality, the dynamic equations of temporal coupled mode theory (TCMT) can be written as (*57*):

$$\frac{d\boldsymbol{a}}{dt} = (j\Omega - \Gamma)\boldsymbol{a} + K^T|s_+\rangle \quad (S.1)$$

$$|s_-\rangle = C|s_+\rangle + D\boldsymbol{a}, \quad (S.2)$$

where both Ω and Γ are $n \times n$ Hermitian matrices, and represent the resonance frequencies and decay rate, respectively. The resonant modes $\boldsymbol{a} = (a_1\ a_2\ ...\ a_n)$ are excited by the incoming waves $|s_+\rangle = (s_{1+}\ s_{2+}\ ...\ s_{n+})^T$ and coupled with the outgoing waves $|s_-\rangle = (s_{1-}\ s_{2-}\ ...\ s_{n-})^T$. Based on the time-reversal symmetry and energy conservation arguments, the coupling matrices K, C and D are not independent but follow the relationship as follows:

$$D^\dagger D = 2\Gamma \quad (S.3)$$

$$K = D \quad (S.4)$$

$$CD^* = -D \quad (S.5)$$

Taking a spatially symmetric system with two modes and two ports as an example, the system can be described as by matrices:

$$\Omega = \begin{bmatrix} \omega_1 & \kappa \\ \kappa & \omega_2 \end{bmatrix} \quad (S.6)$$

$$\Gamma = \begin{bmatrix} \gamma_1 + \gamma_{abs} & \gamma_0 \\ \gamma_0^* & \gamma_2 + \gamma_{abs} \end{bmatrix} \quad (S.7)$$

$$\mathrm{C} = \begin{bmatrix} r_d & t_d \\ t_d & r_d \end{bmatrix} \quad (S.8)$$

$$\mathrm{D} = \begin{bmatrix} d_{11} & d_{12} \\ d_{21} & d_{22} \end{bmatrix} \quad (S.9)$$

where ω_j and γ_j (j = 1,2,3) represent the resonant frequencies and leakage rates of the resonances. κ denotes the near-field coupling factor, γ_0 is the interference of the radiative waves through far-field coupling. Assume that the proposed system is lossless ($\gamma_{abs} = 0$) and possesses a mirror plane symmetry corresponding to ports, from Eq. (S.3), we can obtain that:

$$|d_{11}|^2 + |d_{21}|^2 = 2\gamma_1 \tag{S.10}$$

$$|d_{12}|^2 + |d_{22}|^2 = 2\gamma_2 \tag{S.11}$$

$$|d_{11}||d_{12}|e^{i(\theta_{12}-\theta_{11})} + |d_{21}||d_{22}|e^{i(\theta_{22}-\theta_{21})} = 2|\gamma_0|e^{i\psi} \tag{S.12}$$

where θ_{mn} represents the phase angles of d_{mn} $(m, n = 1,2)$, and ψ represent the phase angle of γ_0. Assume that the system is spatially mirror symmetric to the ports, we get:

$$|d_{11}| = |d_{21}| = \sqrt{\gamma_1} \tag{S.13}$$

$$|d_{12}| = |d_{22}| = \sqrt{\gamma_2} \tag{S.14}$$

$$\sqrt{\gamma_1\gamma_2}\left[e^{i(\theta_{12}-\theta_{11})} + e^{i(\theta_{22}-\theta_{21})}\right] = 2|\gamma_0|e^{i\psi} \tag{S.15}$$

Since the system is mirror symmetric, each mode will decay symmetrically ($\theta_{1j} = \theta_{2j} + 2n\pi$) or anti-symmetrically ($\theta_{1j} = \theta_{2j} + (2n+1)\pi$) into the two ports. When two modes with opposite symmetry coupling, we have $e^{i(\theta_{12}-\theta_{11})} + e^{i(\theta_{22}-\theta_{21})} = 0$. Thus, $\gamma_0 = 0$ and near-field coupling κ should also be zero because the overlap integral of the even and odd modes is zero. This explains the zero values of Hamiltonian Eq. (1) in the main text. When two modes with the same symmetry coupling, we have $e^{i(\theta_{12}-\theta_{11})} = e^{i(\theta_{22}-\theta_{21})}$. Thus, $\gamma_0 = e^{i\psi}\sqrt{\gamma_1\gamma_2}$, where $\psi = |\theta_{12} - \theta_{11}| = |\theta_{22} - \theta_{21}|$ represents the phase difference between the two modes. Only if the scattering matrix C is unitary, by applying the conclusion Eq. (S.5), the symmetric modes will interfere constructively $\left[e^{i(\theta_{12}-\theta_{11})} = e^{i(\theta_{22}-\theta_{21})} = 1, \gamma_0 = \sqrt{\gamma_1\gamma_2}\right]$ or destructively $\left[e^{i(\theta_{12}-\theta_{11})} = e^{i(\theta_{22}-\theta_{21})} = -1, \gamma_0 = -\sqrt{\gamma_1\gamma_2}\right]$. In a more general case where C is not an identity matrix, Fano interference occurs (*57,58*).

Thus, considering the problem of EPs with mode interference, we can use the 2×2 Hamiltonian matrix to explain. The eigenvalues $\widetilde{\Omega}_\pm$ of Hamiltonian $\widehat{H}$ mentioned in the main text [Eq. (1)] can be obtained by solving the following characteristic polynomial:

$$\left|\widehat{H} - \widetilde{\Omega}_\pm \widehat{E}\right| = 0 \tag{S.16}$$

$$\begin{vmatrix} (\omega_1 - i\gamma_1) - \widetilde{\Omega}_\pm & \kappa - i\,\gamma_0 \\ \kappa - i\,\gamma_0 & (\omega_2 - i\gamma_2) - \widetilde{\Omega}_\pm \end{vmatrix} = 0 \tag{S.17}$$

where the $\widehat{E}$ is the unit matrix. The complex eigenvalues $\widetilde{\Omega}_\pm$ can be written as:

$$\tilde{\Omega}_{\pm} = \frac{\omega_1 + \omega_2}{2} - i\frac{\gamma_1 + \gamma_2}{2} \pm \sqrt{\left(\frac{\omega_1 - \omega_2}{2} - i\frac{\gamma_1 - \gamma_2}{2}\right)^2 + (\kappa - i\,\gamma_0)^2}$$
$$= \frac{\omega_1 + \omega_2}{2} - i\frac{\gamma_1 + \gamma_2}{2} \pm \sqrt{(\kappa - i\,\gamma_0)^2 - \left(\frac{\gamma_2 - \gamma_1}{2} - i\frac{\omega_1 - \omega_2}{2}\right)^2}$$
$$= \omega_{ave} - i\gamma_{ave} \pm \sqrt{(\kappa - i\,\gamma_0)^2 - \left(-\Delta\gamma - i\Delta\omega\right)^2} \quad (\mathrm{S.18})$$

where $\omega_{ave} = (\omega_1 + \omega_2)/2$, $\gamma_{ave} = (\gamma_1 + \gamma_2)/2$, $\Delta\omega = (\omega_1 - \omega_2)/2$, $\Delta\gamma = (\gamma_1 - \gamma_2)/2$. And the corresponding eigenvectors can be written as:

$$\tilde{\upsilon}_{\pm} = -\frac{\left(\Delta\omega - i\Delta\gamma\right) \mp \sqrt{(\kappa - i\,\gamma_0)^2 + \left(\Delta\omega - i\Delta\gamma\right)^2}}{\kappa - i\,\gamma_0}$$
$$= -\frac{\Delta\tilde{\Omega}}{\tilde{\kappa}} \mp \sqrt{\left(\frac{\Delta\tilde{\Omega}}{\tilde{\kappa}}\right)^2 + 1} \quad (\mathrm{S.19})$$

where $\Delta\tilde{\Omega} = \Delta\omega - i\Delta\gamma$, $\tilde{\kappa} = \kappa - i\,\gamma_0$. Thus, the corresponding eigenstates can be written as $(1 \quad \tilde{\upsilon}_{\pm})^T$. It can be concluded that EP occurs when the square root term of Eq. (S.3) and Eq. (S.4) are both 0, where two complex eigenvalues coalesce. The conditions of EP are:

$$\begin{cases} \kappa = \pm\Delta\gamma \\ \Delta\omega = \mp\,\gamma_0 \end{cases} \quad (\mathrm{S.20})$$

S2. Multipole decomposition

The multipole decomposition method is performed to calculate the contributions of different multipoles in scattering spectra. The multipoles are generated by the induced polarization current in dielectric scatters. The induced current density is defined in the dielectric as follows:

$$\boldsymbol{J} = i\omega\varepsilon_0(\varepsilon_r - 1)\boldsymbol{E}, \quad (\mathrm{S.21})$$

where ω is the angular frequency, ε_0 is the vacuum permittivity, ε_r is the relative permittivity of the dielectric, and E is the electric field. Here, the time dependence is defined by $e^{i\omega t}$. The scattered power defined by various multipoles can be calculated as (*59,60*):

$$P_{sca} = \frac{k^4}{12\pi\varepsilon_0^2 c\mu_0}\left|P_\alpha + \frac{ik}{c}T_\alpha^{(e)}\right|^2 + \frac{k^4}{12\pi\varepsilon_0 c}\left|m_\alpha + \frac{ik}{c}T_\alpha^{(m)}\right|^2$$
$$+ \frac{k^6}{160\pi\varepsilon_0^2 c\mu_0}\left|\hat{Q}_{\alpha\beta}^{(e)}\right|^2 + \frac{k^6}{160\pi\varepsilon_0 c}\left|\hat{Q}_{\alpha\beta}^{(m)}\right|^2 + \cdots \quad (\mathrm{S.22})$$

where μ_0 is the vacuum permeability, $k = \omega/c$ is the wave number in vacuum. The general expressions for multipole in Cartesian coordinate are given below:

Electric dipole (ED) moment:

$$P_\alpha = \frac{i}{\omega}\int J_\alpha \, dv. \quad (S.23)$$

Toroidal electric dipole (TED) moment:

$$T_\alpha^{(e)} = \frac{1}{10}\int |(J \cdot r) r_\alpha - 2r^2 J_\alpha| dv. \quad (S.24)$$

Magnetic dipole (MD) moment:

$$m_\alpha = \frac{1}{2}\int (r \times J)_\alpha dv. \quad (S.25)$$

Toroidal magnetic dipole (TMD) moment:

$$T_\alpha^{(m)} = \frac{i\omega}{20}\int r^2 (r \times J)_\alpha dv. \quad (S.26)$$

Electric quadrupole (EQ) moment:

$$\hat{Q}_{\alpha\beta}^{(e)} = \frac{i}{\omega}\int \left| r_\alpha J_\beta + r_\beta J_\alpha - \frac{2}{3}\delta_{\alpha\beta}(r \cdot J) \right| dv. \quad (S.27)$$

Magnetic quadrupole (MQ) moment:

$$\hat{Q}^{(m)} = \frac{1}{3}\int \left[(r \times J)_\alpha r_\beta + (r \times J)_\beta r_\alpha\right] dv, \quad (S.28)$$

where $\alpha, \beta = x, y, z$ represent the directions in Cartesian coordinates. r is the position vector, $\delta_{\alpha\beta}$ is the delta Kronecker symbol. The scattering cross-section is obtained via the normalization of the scattered power to the incident energy flux:

$$\sigma_{sca} = 2\sqrt{\frac{\mu_0}{\varepsilon_0}}\frac{P_{sca}}{|E_{inc}|^2} \quad (S.29)$$

S3. Quasi-normal modes and Fano Fitting

Generally, such an open system has resonances with different frequencies and decay rates. In the frequency domain, these resonances lead to various peaks in the spectrum of observable quantity, for example, cross sections. Therefore, the electromagnetic fields in the resonator can be considered as a sum over a set of QNMs (*50*):

$$\boldsymbol{E}(\boldsymbol{r}, t) = Re\left\{\sum_m A_m(t)\widetilde{\boldsymbol{E}}_m(\boldsymbol{r})e^{-i\Omega_m t}e^{-\Gamma_m t/2}\right\} \quad (S.30)$$

where A_m is the excitation coefficient, $\widetilde{\boldsymbol{E}}_m(\boldsymbol{r})$ is the spatial profile of QNMs, Ω_m is the resonance frequency and Γ_m is the damping rate. Thus, the QNMs are time-harmonic fields with a complex frequency:

$$\widetilde{\omega}_m = \Omega_m - i\Gamma_m/2 \quad (S.31)$$

The real parts mean the positions of resonance peaks, while the imaginary parts mean the full width at half of the maximum (FWHM) of resonance peaks.

In this way, the QNMs of an open system can be calculated by solving the time harmonics source-free Maxwell's equations, and they should also satisfy the outgoing-wave boundary conditions:

$$\begin{bmatrix} 0 & i\varepsilon^{-1}(\boldsymbol{r},\tilde{\omega}_m)\nabla\times \\ -i\mu^{-1}(\boldsymbol{r},\tilde{\omega}_m)\nabla\times & 0 \end{bmatrix}\begin{bmatrix} \tilde{\boldsymbol{E}}_m(\boldsymbol{r}) \\ \tilde{\boldsymbol{H}}_m(\boldsymbol{r}) \end{bmatrix} = \tilde{\omega}_m \begin{bmatrix} \tilde{\boldsymbol{E}}_m(\boldsymbol{r}) \\ \tilde{\boldsymbol{H}}_m(\boldsymbol{r}) \end{bmatrix} \quad (\mathrm{S}.32)$$

where $\varepsilon(\boldsymbol{r},\tilde{\omega}_m)$ and $\mu(\boldsymbol{r},\tilde{\omega}_m)$ are space and frequency-dependent permittivity and permeability tensors of the resonator and surrounding background. And $\tilde{\omega}_m$ are eigenvalues, $\begin{bmatrix} \tilde{\boldsymbol{E}}_m(\boldsymbol{r}) \\ \tilde{\boldsymbol{H}}_m(\boldsymbol{r}) \end{bmatrix}$ are eigenvectors. The equation (S.32) can be rewritten as:

$$\widehat{\boldsymbol{H}}(\boldsymbol{r},\tilde{\omega}_m)\tilde{\boldsymbol{\Psi}}_m(\boldsymbol{r}) = \tilde{\omega}_m\tilde{\boldsymbol{\Psi}}_m(\boldsymbol{r}) \quad (\mathrm{S}.33)$$

The orthogonality of QNMs' basis is proved in (*61-63*) with Lorentz reciprocity theorem, so $\widehat{\boldsymbol{H}}$ is diagonalizable and the scattered field can be viewed as a sum of modes:

$$\boldsymbol{\Psi}_{sca}(\boldsymbol{r},\omega) = \sum_m \alpha_m(\omega)\,\tilde{\boldsymbol{\Psi}}_m(\boldsymbol{r}) \quad (\mathrm{S}.34)$$

where $\alpha_m(\omega)$ is the excitation coefficient by the incident wave, the eigenvectors $\tilde{\boldsymbol{\Psi}}_m(\boldsymbol{r})$ will not depend on the excitation. It allows the reconstruction of the scattering fields from the sum of modes.

When considering the scattering of an incident field by a resonator, the permittivity $\varepsilon(\boldsymbol{r},\omega)$ of the total system (resonator and background) can be decomposed as $\varepsilon(\boldsymbol{r},\omega) = \varepsilon_b(\boldsymbol{r},\omega) + \Delta\varepsilon(\boldsymbol{r},\omega)$, where $\varepsilon_b(\boldsymbol{r},\omega)$ represents a background permittivity and $\Delta\varepsilon(\boldsymbol{r},\omega)$ is null everywhere except in the resonant structure. In the absence of the resonator, the background electromagnetic fields $\begin{bmatrix} \boldsymbol{E}_b \\ \boldsymbol{H}_b \end{bmatrix}$ satisfy the following equations:

$$\begin{cases} \nabla\times\boldsymbol{E}_b = i\omega\mu(\boldsymbol{r},\omega)\boldsymbol{H}_b \\ \nabla\times\boldsymbol{H}_b = -i\omega\varepsilon_b(\boldsymbol{r},\omega)\boldsymbol{E}_b + \boldsymbol{J}(\boldsymbol{r},\omega) \end{cases} \quad (\mathrm{S}.35)$$

where $\boldsymbol{J}(\boldsymbol{r},\omega)$ is the current distribution of the source generating the background field. In the presence of resonator, the total fields $\begin{bmatrix} \boldsymbol{E} \\ \boldsymbol{H} \end{bmatrix}$ satisfy the following equations:

$$\begin{cases} \nabla\times\boldsymbol{E} = i\omega\mu(\boldsymbol{r},\omega)\boldsymbol{H} \\ \nabla\times\boldsymbol{H} = -i\omega\varepsilon(\boldsymbol{r},\omega)\boldsymbol{E} + \boldsymbol{J}(\boldsymbol{r},\omega) \end{cases} \quad (\mathrm{S}.36)$$

where the source term $\boldsymbol{J}(\boldsymbol{r},\omega)$ is the same. The scattered fields can be calculated by $\begin{bmatrix} \boldsymbol{E}_s \\ \boldsymbol{H}_s \end{bmatrix} = \begin{bmatrix} \boldsymbol{E} \\ \boldsymbol{H} \end{bmatrix} - \begin{bmatrix} \boldsymbol{E}_b \\ \boldsymbol{H}_b \end{bmatrix}$, which satisfy the following Maxwell's equations:

$$\begin{cases} \nabla \times \boldsymbol{E}_s = i\omega\mu(\boldsymbol{r},\omega)\boldsymbol{H}_s \\ \nabla \times \boldsymbol{H}_s = -i\omega\varepsilon_b(\boldsymbol{r},\omega)\boldsymbol{E}_s - i\omega\Delta\varepsilon(\boldsymbol{r},\omega)\boldsymbol{E}_b \end{cases} \quad (\mathrm{S.37})$$

The Eq. (S.37) implies that the scattered fields can be considered as those fields radiated by a current-source distribution $-i\omega\Delta\varepsilon(\boldsymbol{r},\omega)\boldsymbol{E}_b$ in the presence of the resonator. This current-source is a well-defined quantity, determined entirely by the incident fields and the specified background.

The absorbed (P_{abs}) and scattered (P_{sca}) powers can be determined analytically using the Poynting theorem (*64*) as follows:

$$\mathrm{P}_{abs} = \frac{\omega}{2}\iiint_{V_r} Im(\varepsilon)|\boldsymbol{E}_s + \boldsymbol{E}_b|^2 d^3\boldsymbol{r} \quad (\mathrm{S.38})$$

$$\mathrm{P}_{sca} = \frac{1}{2}\iint_{\Sigma_r} Re(\boldsymbol{E}_s \times \boldsymbol{H}_s^*) \cdot d\boldsymbol{S} \quad (\mathrm{S.39})$$

where Σ_r is a closed surface enclosed V_r which surrounding the resonant structure. While the Ref. (*62*) finds that, based on numerical tests, greater accuracy for P_{sca} is reached when a small number of the QNMs if P_{sca} is expressed as a volume integral instead of using a surface integral. By applying the Poynting theorem and Lorentz reciprocity theorem (*50*), one can obtain that:

$$\mathrm{P}_{sca} = -\frac{\omega}{2}\iiint_{V_r} Im(\varepsilon_b)|\boldsymbol{E}_s|^2 d^3\boldsymbol{r} - \frac{\omega}{2}\iiint_{V_r} Im[\Delta\varepsilon\boldsymbol{E}_s^* \cdot (\boldsymbol{E}_s + \boldsymbol{E}_b)] d^3\boldsymbol{r} \quad (\mathrm{S.40})$$

Thus, the summation of the scatter power P_{sca} in Eq. (S.40) and absorbed power P_{abs} in Eq. (S.38) gives the extinction power as:

$$\mathrm{P}_{\mathrm{ext}} = \frac{\omega}{2}\iiint_{V_r} Im[\Delta\varepsilon\boldsymbol{E}_b^* \cdot (\boldsymbol{E}_s + \boldsymbol{E}_b)] d^3\boldsymbol{r} + \frac{\omega}{2}\iiint_{V_r} Im(\varepsilon_b)[|\boldsymbol{E}_s + \boldsymbol{E}_b|^2 - |\boldsymbol{E}_s|^2] d^3\boldsymbol{r} \quad (\mathrm{S.41})$$

When the resonator is placed in a lossless background where $Im(\varepsilon_b) = 0$, the second term in Eq. (S.41) becomes zero, reducing Eq. (S.41) to the specified form as proposed in Ref. (*50*):

$$\mathrm{P_{ext}} = \frac{\omega}{2}\iiint\limits_{V_r} Im[\Delta\varepsilon \boldsymbol{E}_b^* \cdot (\boldsymbol{E}_s + \boldsymbol{E}_b)] d^3\boldsymbol{r}$$
$$= \frac{\omega}{2}\iiint\limits_{V_r} Im(\Delta\varepsilon \boldsymbol{E}_b^* \boldsymbol{E}_s) d^3\boldsymbol{r} + \frac{\omega}{2}\iiint\limits_{V_r} Im(\Delta\varepsilon \boldsymbol{E}_b^* \boldsymbol{E}_b) d^3\boldsymbol{r} \quad (S.42)$$

The extinction cross section can be obtained by the definition $\sigma_{\mathrm{ext}} = \mathrm{P_{ext}}/\mathrm{S_0}$:

$$\sigma_{ext} = \frac{\omega}{2S_0}\int Im[\Delta\varepsilon \boldsymbol{E}_b^* \cdot (\boldsymbol{E}_s + \boldsymbol{E}_b)]$$
$$= \frac{\omega}{2S_0}\iiint\limits_{V_r} Im(\Delta\varepsilon \boldsymbol{E}_b^* \boldsymbol{E}_s) d^3\boldsymbol{r} + \frac{\omega}{2S_0}\iiint\limits_{V_r} Im(\Delta\varepsilon \boldsymbol{E}_b^* \boldsymbol{E}_b) d^3\boldsymbol{r} \quad (S.43)$$

where $\mathrm{S_0}$ represents the time-averaged Poynting vector of the incident plane wave. The first term of Eq. (S.43) denotes for resonant term, while the second term denotes for non-resonant (background) term. From Eq. (S.34), we know that the scattered field $\boldsymbol{E}_s$ can be rewritten as a summation of QNMs. Thus, the Eq. (S.43) can be rewritten as:

$$\sigma_{ext} = \sum_m \sigma_m + \sigma_{\mathrm{non}} \quad (S.44)$$

where $\sigma_m = \frac{\omega}{2S_0}\iiint_{V_r} Im\left(\Delta\varepsilon \boldsymbol{E}_b^* \alpha_m(\omega) \widetilde{\boldsymbol{E}}_m(\boldsymbol{r})\right) d^3\boldsymbol{r}$ denotes for the resonant term, and $\sigma_{\mathrm{non}} = \frac{\omega}{2S_0}\iiint_{V_r} Im(\Delta\varepsilon \boldsymbol{E}_b^* \boldsymbol{E}_b) d^3\boldsymbol{r}$ denotes for the non-resonant term.

Each QNM response is fitted using the following Fano formula (*65-67*):

$$\sigma = \mathrm{I}\frac{(\Omega + q)}{(\Omega^2 + 1)} \quad (S.45)$$

where q is the Fano asymmetry parameter, $\Omega = (\omega - \omega_0)/2\Gamma_0$ where Γ_0 and ω_0 correspond to the half-width and frequency of the resonance, respectively. To gain insight into the region of interest, we employ a set of parameters for all three resonances. This approach involves three sets of (ω_0, Γ_0, q, I) as an initial approximation of parameters to fit the experimental spectrum using the sum of three Fano contours. **Fig. S5** provides an illustrative example of an extinction spectrum fitted using the QNMs method. Each QNMs can be fitted by the Fano profiles (solid red, green and orange lines in **Fig. S5**), and the total extinction cross section can be viewed as the summation of all QNMs (solid blue line in **Fig. S5**) owing to the orthogonality of QNMs theory. In this way, the intricate spectra can be separated independently by Fano profiles, thereby enabling the detection of the eigenfrequency shift.

S4. TCMT approximation in two-mode system

In this section, we discuss the mechanism of a two-mode coupling system in a dielectric ring-shaped resonator. As shown in the previous section, EPs are realized not in real but in parametric space by tuning the coupling and frequency detuning. We make an approximation with the Hamiltonian in the TCMT model and obtain the corresponding fitted coupling values shown in **Fig. S2**. We extract the uncoupled complex eigenfrequencies $\omega_{\mathrm{j}} - i\gamma_{\mathrm{j}}$ ($\mathrm{j} = 1{,}2$), and the corresponding eigenvalues $\tilde{\Omega}_{\pm}$ obtained in the COMSOL simulation. We find that the varying $R_{\mathrm{in}}/R_{\mathrm{out}}$ has an obvious influence on the coupling (**Fig. S2A**), for each fixed $R_{\mathrm{in}}/R_{\mathrm{out}}$, the coupling coefficient will stay almost constant whatever $\Delta R/h$ changes. The change in $\Delta R/h$ will mainly affect the frequency detuning $\Delta\omega$ instead of the coupling (**Fig. S2B**). The strength of near-field coupling κ can be approximately considered to be the overlap integral between the electric fields of eigenmodes (*8*). The overlap integral over the removed volume due to changes in height is negligible, whereas the overlap integral of the eigenmodes becomes significant when the air hole expands. So, changing the aspect ratio $\Delta R/h$ primarily adjusts the eigenfrequency detuning $\Delta\omega$, while changing the radius ratio $R_{\mathrm{in}}/R_{\mathrm{out}}$ primarily adjusts the coupling strength κ. And the similar simulated results have also been published in (*7-9*). Our theoretical approximation has a good agreement with the simulated results, showing the possibility of our analytical model to approach EP by manipulating the parameter space.

S5. Effect of material loss

In this section, we discuss the effect of material losses on both eigenvalue and cross-section problems. By extending the 2×2 Hamiltonian in **S1** with material loss to

$$\hat{H} = \begin{pmatrix} \omega_1 & \kappa \\ \kappa & \omega_2 \end{pmatrix} - \underbrace{i\begin{pmatrix} \gamma_1 & \gamma_0 \\ \gamma_0 & \gamma_2 \end{pmatrix}}_{\text{Radiation loss}} - \underbrace{i\begin{pmatrix} \gamma_{\mathrm{abs}} & 0 \\ 0 & \gamma_{\mathrm{abs}} \end{pmatrix}}_{\text{Material loss}} \tag{S.46}$$

where γ_{abs} represents the material loss. The eigenvalues (S.18) can be generalized as:

$$\tilde{\Omega}_{\pm} = \omega_{ave} - i\gamma_{ave} - i\gamma_{abs} \pm \sqrt{(\Delta\omega - i\Delta\gamma)^2 + (\kappa - i\,\gamma_0)^2} \tag{S.47}$$

The off-diagonal matrix of material loss won't change the condition of EPs (Because the square root term of Eq. (S.47) doesn't change), but it will increase the imaginary parts of each mode. The corresponding simulated real eigenfrequencies and quality factor (Q-factor) are illustrated in **Fig. S4A** and **Fig. S4B**. With the increasing of material losses from $\delta = 0$ to $\delta = 1.8 \times 10^{-2}$, the position of real eigenfrequencies don't change (**Fig. S4A**), but the Q-factors decrease (**Fig. S4B**), especially in the vicinity of Q-BICs [Because the absorption Q-factor limits the total Q-factor, this phenomenon has been discussed well in previous Q-BICs related papers, such as (*8*)]. Excited by the linear polarized plane electromagnetic

wave, the samples with material losses will have absorption cross-sections. In our experimental samples, the material loss tangent is $\delta = 1.8 \times 10^{-4}$, shown in **Fig. S4D**. The influence of absorption cross-section is neglectable. However, with the increase of material losses, the influence of absorption cross section will become more pronounced (**Fig. S4C-F**).

S6. Mode symmetry analysis

In a dielectric cylindrical resonator, the mode symmetry is primarily determined by its rotational and mirror symmetry, characterized by the symmetry group D_{nh} (*51,52*). The $D_{\infty h}$ group represents an infinite-order dihedral symmetry. Each mode can be classified by its azimuthal number m, which defines the rotational properties of field distribution around the cylinder axis, and by its parity (odd or even), indicating how the mode behaves under inversion. Modes are grouped into different irreducible representations depending on these symmetries. The modes with $m = 0$ can be additionally classified into transverse electric (TE) and transverse magnetic (TM) modes. Only modes from the same irreducible representation can couple under transformations of the resonator, preserving its symmetry. The high symmetry of the cylinder simplifies mode analysis, allowing for a clear understanding of the electromagnetic field distribution and resonance behavior.

S7. EP at coupling transition

For a two-mode system, the strong mode coupling regime is characterized by the anti-crossing of real frequencies and crossing of imaginary frequencies, while the feature of weak mode coupling regime is the crossing of real frequencies with the repulsion of imaginary frequencies. As the critical state between strong and weak mode coupling regimes, the real and imaginary parts of the exceptional point merge (*15-17*).

As mentioned previously, altering radius ratio of the resonator can mainly change the mode coupling, and the varying aspect ratio can locate the position of resonance anti-crossing or crossing, resulting in the transition from PT-symmetry to broken-PT phases. **Fig. S7A** and **Fig. S7B** show the simulated complex eigenfrequency surfaces calculated with COMSOL Multiphysics, where the EP appears at the point of strong-weak coupling transition marked by the golden star. **Fig. S7C-E** show the corresponding complex eigenfrequency characteristics from a strong coupling regime to a weak coupling regime.

S8. Parity-time symmetry with Multipole EP

Learned from the equation (S.20), PT symmetry can be realized by optimizing the detuning and coupling. In the proposed dielectric ring-shaped resonator, PT symmetry can

be achieved by changing the structural parameters, which is related to the change in both system coupling and detuning. Calculated by the eigenfrequency solver in COMSOL Multiphysics, **Fig. S8A** and **Fig. S8B** show the transition from PT-symmetry to broken-PT, ED and MD types of EPs appear at the critical state where PT phase transition occurs.

Noted that $R_{\text{in}}/R_{\text{out}}$ in **Fig. S8A** is varied from 0.476 to 0.521, ensuring the complex eigenfrequencies of the two ED modes coinciding at different $\Delta R/h$ (refer to **Table. S1**). At $\Delta R/h < 0.4524$, the weak coupling strength is not large enough to compensate for the loss difference, indicating the system is in the PT-broken phase. The two modes locate at the same frequency but with distinct levels of losses, leading to the differences in electric field distributions. At $\Delta R/h = 0.4524$, both real and imaginary parts of the eigenfrequency coalesce. The electric field distributions of the two eigenmodes are the same, since the coalescence of the eigenvector is also at EP. At $\Delta R/h > 0.4524$, the system is in the PT-symmetry phase, where two eigenmodes with the equal losses are located on two branches of the real frequency. Also, the field distributions of the two eigenmodes are distorted after the EP, representing the exchange of the eigenmode at EP. The same case happens in **Fig. S8B**, where $R_{\text{in}}/R_{\text{out}}$ it is varied from 0.272 to 0.315, ensuring the complex eigenfrequencies of the two MD modes coincide at different $\Delta R/h$ (refer to **Table. S2**). MD type of EP appears when $\Delta R/h = 0.4095$, $R_{\text{in}}/R_{\text{out}} = 0.2945$.

It can be concluded that EPs manifest at the coalescence between modes with identical azimuthal numbers. The left panel of **Fig. S8C** illustrates the existence of multifaceted EPs with varying azimuthal numbers within the structural parameter space. The right panel of **Fig. S8C** illustrates the azimuthal-component of electric field distribution of various EPs. It is observed that the quality-factors of EPs exhibit an order-level enhancement when the azimuthal number is 2. This phenomenon can be attributed to the multipolar conversion, which shares similarities with the behavior observed in Q-BICs.

S9. Electric/ Magnetic dipole-contributed EPs in scattering spectra

We investigate the multipole behavior in the evolution of the scattering cross sections in relation to the structural configuration around electric (see **Fig. S8A**) and magnetic (see **Fig. S8B**) dipole-contributed EPs. In **Fig. S9A-C**, the excitation of odd modes (ED, MQ, EO, etc.) with all azimuthal numbers can be achieved under the TE plane wave. **Fig. S9A** illustrates that the scattering dark state (i.e. anapole state) is present between the two ED modes, and will coalesce at the position of the electric dipole EP. **Fig. S9B-C** illustrates that the higher-order multipolar will contribute more than dipole radiation, which will become less prominent due to the destructive interference. Furthermore, it also demonstrates that the

quadrupole radiation begins to increase at EP and reaches its maximum at Q-BICs. **Fig. S9D-F** shows a comparable phenomenon to that observed in **Fig. S9A-C** under the TM plane wave, in this case, the even modes (MD, EQ, MO, etc.) with all azimuthal numbers excited. We also study the amplitude (ED in **Fig. S10A** and MD in **Fig. S10C**) and phase (ED in **Fig. S10B** and MD in **Fig. S10D**)of electric and magnetic dipole in the aforementioned two cases. It is demonstrated that the dipole resonances exhibit an identical amplitude but an out-of-phase relationship in the respective anapole states.

Supplementary Figures

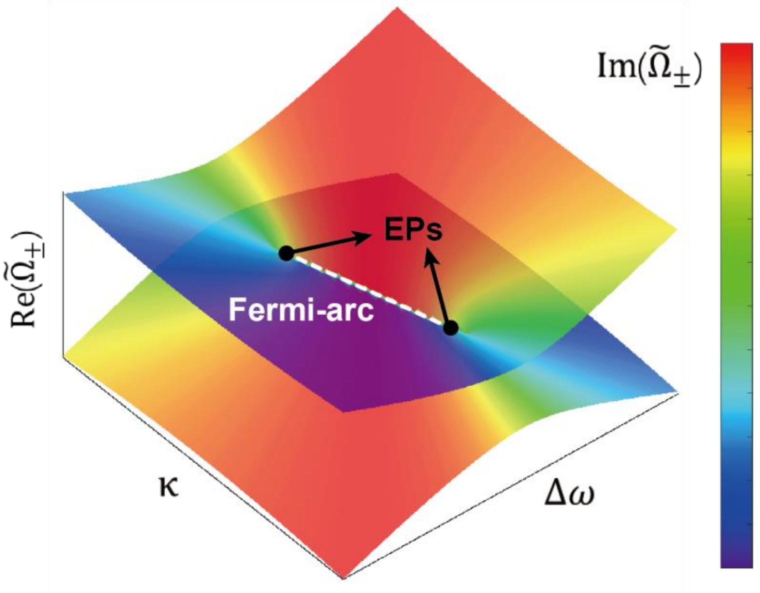

Fig. S1. TCMT prediction of the EPs within parameter space. Complex Eigenvalues of two-mode system [Eq. (S.18)] in terms of the mode coupling κ and system detuning $\Delta\omega$. Paired EPs connected by the Fermi-arc can be predicted in the positions of Eq. (S.18). It can be concluded that EPs can be freely tailored by controlling the mode coupling can system detuning, regardless of the structure.

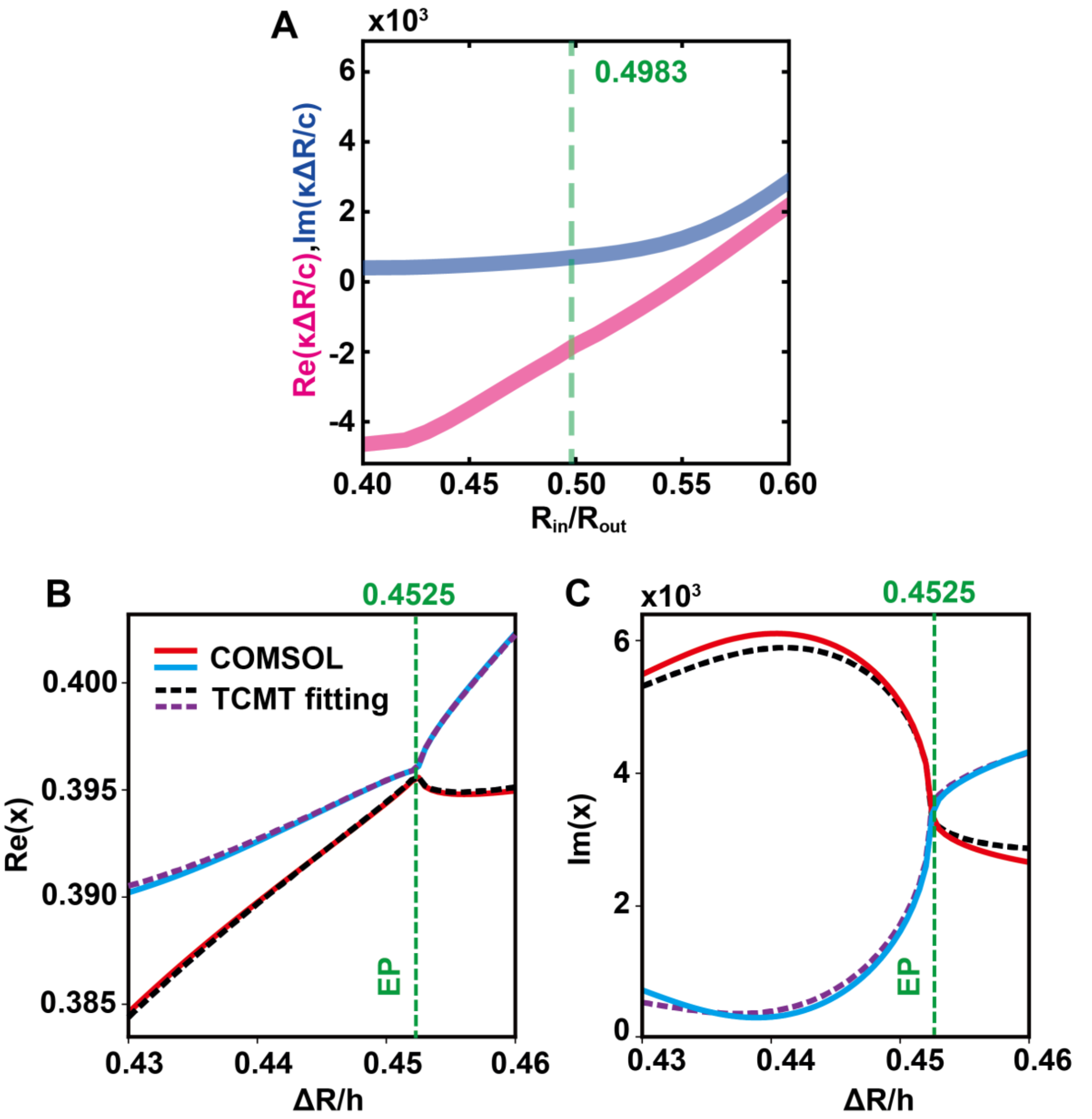

Fig. S2. TCMT approximation of two-mode coupling system: (A) The complex values of the coupling coefficient fitted by the Hamiltonian as a function of radius ratio $R_{\text{in}}/R_{\text{out}}$. The shaded pink and blue curves represent the real and imaginary parts of the fitted coupling coefficient, respectively. The real **(B)** and imaginary **(C)** parts of size parameter $x = \omega\,\Delta R/c$ when $R_{\text{in}}/R_{\text{out}} = 0.4983$ as a function of aspect ratio $\Delta R/h$. The simulated results are illustrated by solid lines (red and blue), and TCMT approximately fitted results are illustrated by dashed lines (black and purple). EP appears at $\Delta R/h = 0.4525$.

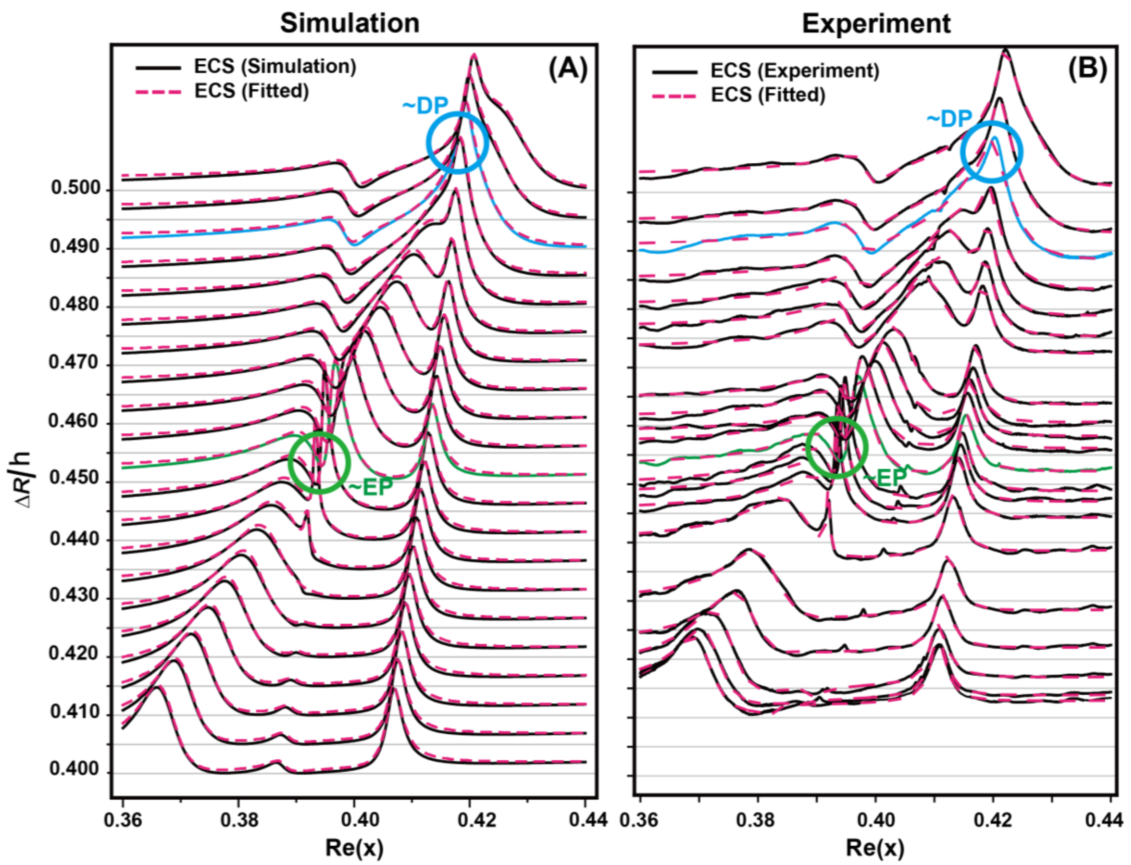

Fig. S3. Evolution of the extinction cross-section in simulation and experiment. (A) In the simulation, the R_{in}/R_{out} is fixed at 0.4983, and the evolution of extinction cross sections with various $\Delta R/h$ shows an excellent agreement with the simulation curves (black solid lines) and the theoretical fitted results (red dashed lines). **(B)** In the experiment, the R_{in}/R_{out} is fixed around 0.498, and the scattering cross sections with various $\Delta R/h$ are not as flat as the simulation results due to the scattering losses from the defect in sample fabrication. The measured curves (black solid lines) and the theoretical fitted results (red dashed lines) are at the position of corresponding $\Delta R/h$. The green and blue circles highlight the positions of EP and DP in the simulation and experiment, respectively.

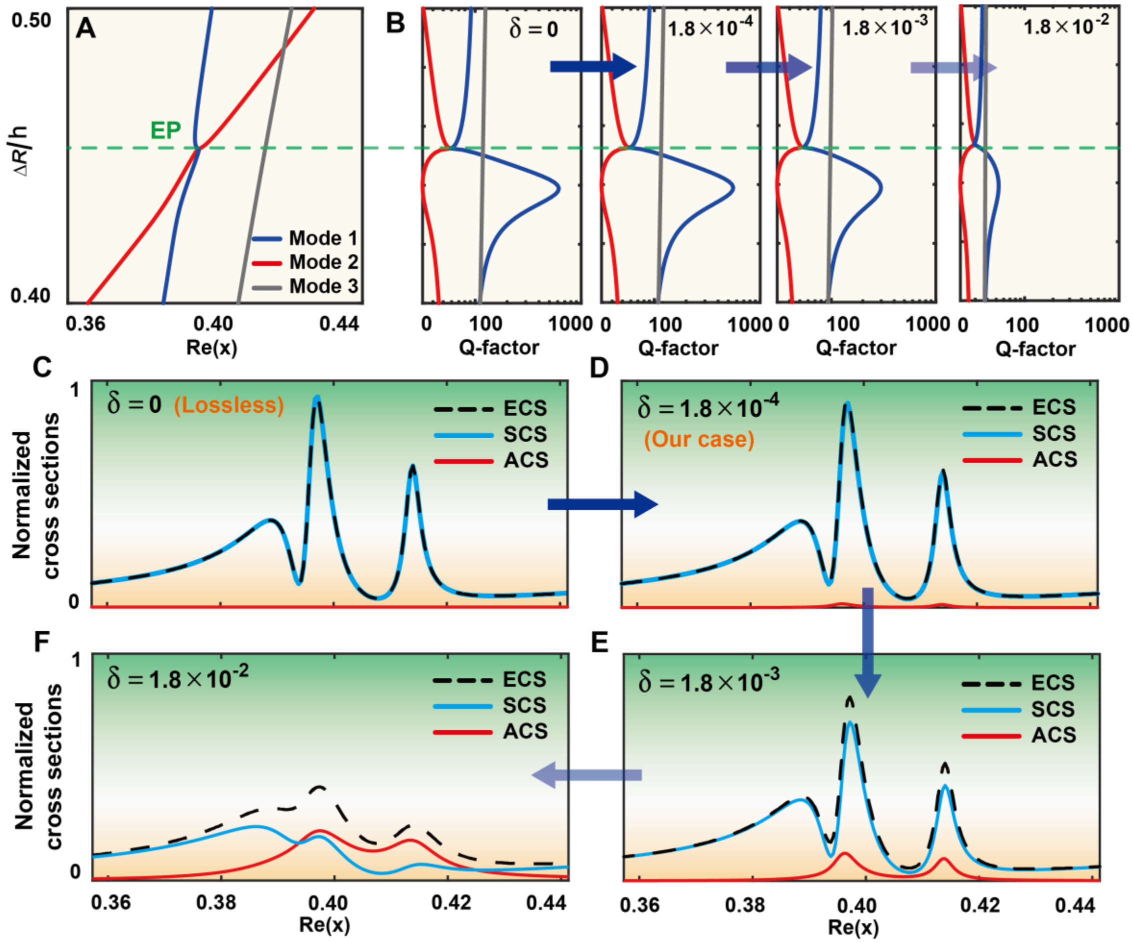

Fig. S4. Effect of the material losses at EP. The evolution of the **(A)** real eigenvalues and the **(B)** quality-factors with varying material loss tangent δ for three modes, represented by distinct colors. EP is indicated by the green dashed line. **(C-F)** The corresponding cross-sections with different material losses. **(C)** In lossless system ($\delta = 0$), the normalized cross-sections - Extinction cross section (ECS), Scattering cross section (SCS) and Absorption cross section (ACS) - are plotted at the EP configuration. **(D)** same panel for the system where $\delta = 1.8 \times 10^{-4}$, matching the value used in experimental samples. **(E)** same panel for the system where $\delta = 1.8 \times 10^{-3}$. **(F)** same panel for the system where $\delta = 1.8 \times 10^{-2}$.

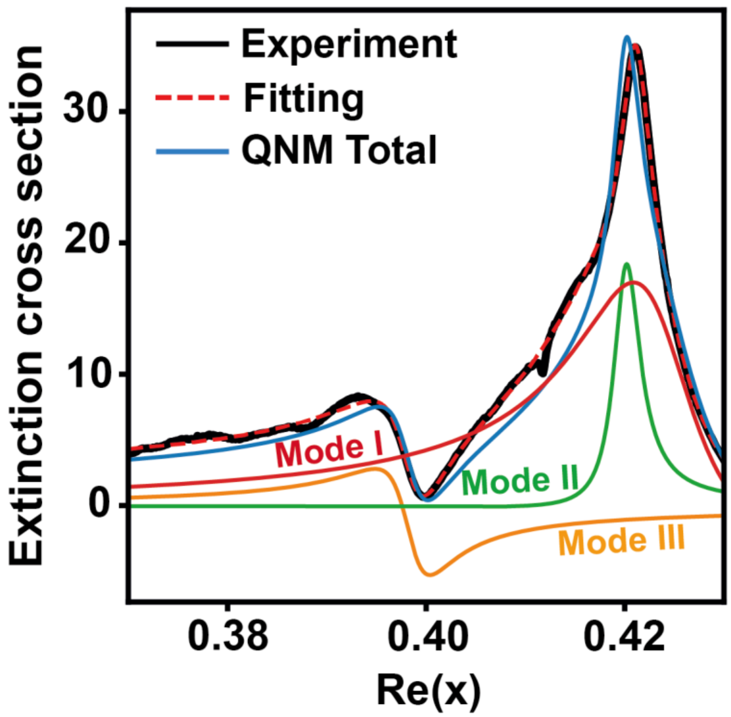

Fig. S5. QNMs fitting of the extinction cross section with $\Delta R/h = 0.4936$. The black solid line represents the experimental extinction cross section, the red, green and orange solid lines represent the spectra contributions of main three QNMs, respectively. The blue solid line is the summation of three QNMs (Eq. S.43), the red dashed line is the fitted results of experimental spectra using parameters from QNMs.

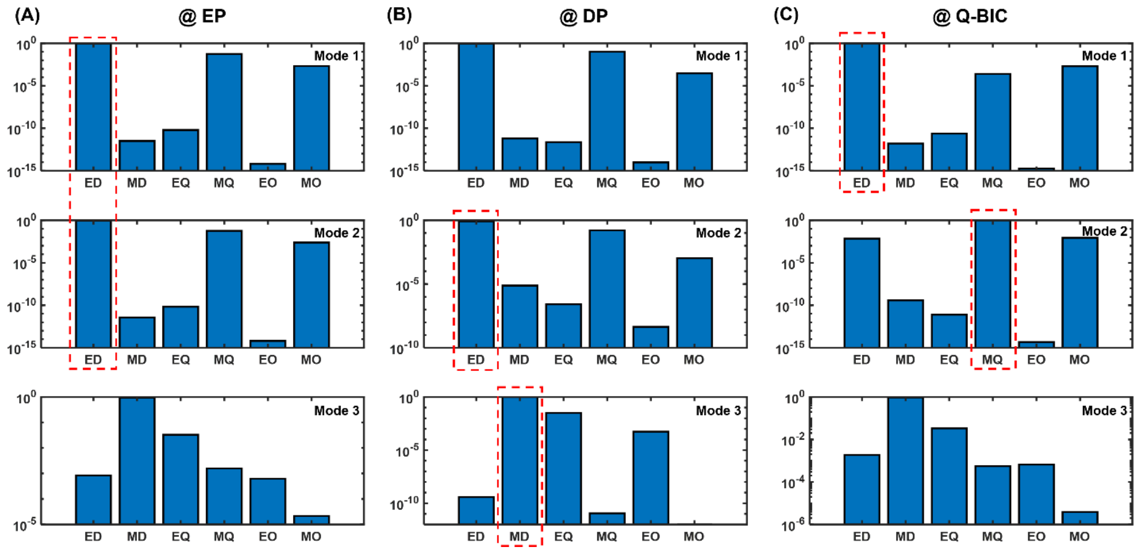

Fig. S6. Proportion of the multipoles of different modes at exceptional point (EP), diabolic point (DP) and quasi-bound state in the continuum (Q-BIC). Note that the numbering of the modes refers to **Fig. 4**, and the y-axis represents the percentage of each multipole (log scale). **(A)** At EP, the mode 1 and mode 2 (with the same principal multipole, i.e. ED, indicated by the red dashed frame) coalesce, resulting in the scattering dark state. **(B)** At DP, the mode 2 (with the principal multipole ED) and mode 3 (with the principal multipole ED) indicated by the red dashed frames merge. Multipoles with opposite symmetry merge won't interfere in the far-field, resulting in the superposition of the resonance peaks. **(C)** At Q-BIC, the mode 1 couples with the mode 2, the destructive interference results in the higher-order multipole (from ED to MQ) conversion at mode 2 and the super radiation at mode 1.

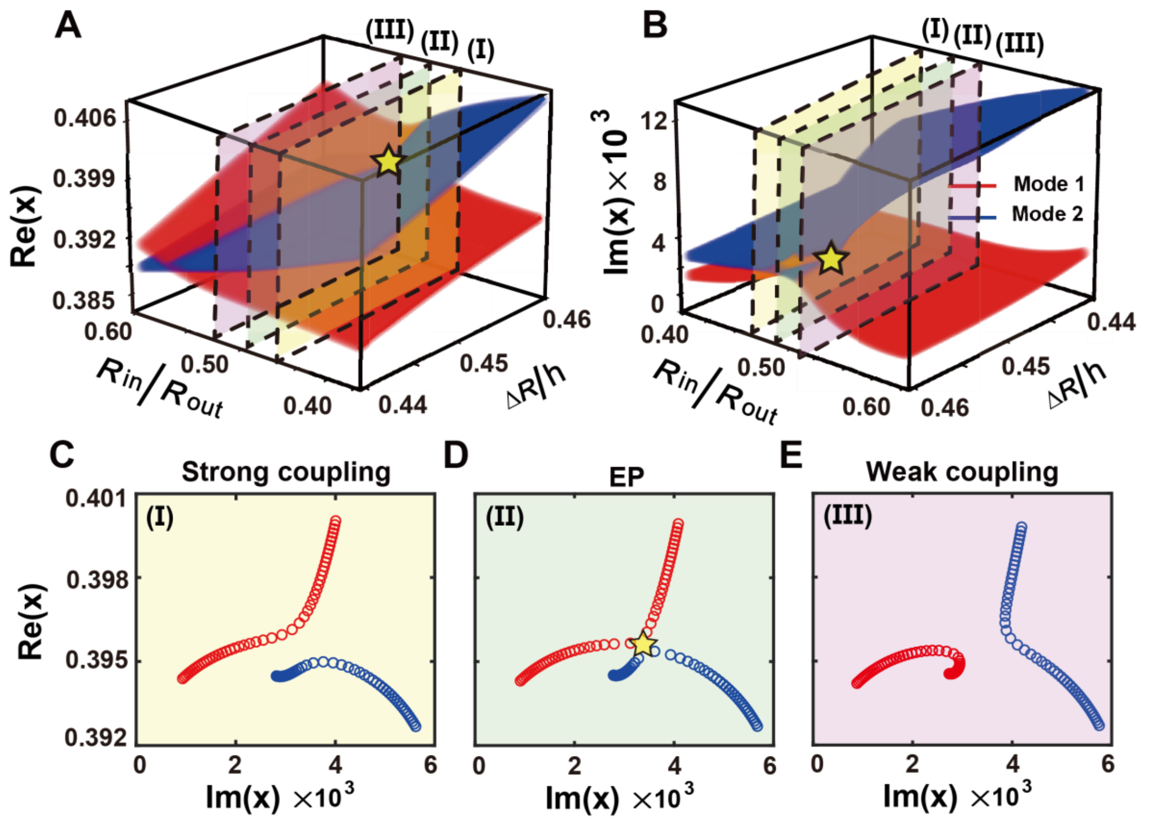

Fig. S7. Exceptional point in a non-Hermitian system. Evolution of the real **(A)** and imaginary **(B)** parts of the eigenfrequencies, the golden star where both real imaginary parts coalesce indicates the position of EP. These panels illustrate the topological characteristic of the eigenfrequency surfaces around an EP. **(C-E)** Eigenfrequency dependencies between real parts and imaginary parts. Owing to the presence of the exceptional point, the parameter space is separated into strong and weak coupling regimes: **(C)** Strong coupling regime ($R_{\rm in}/R_{\rm out} = 0.497$) characterized by the resonance repulsion in the real frequencies, whereas crossing in the imaginary frequencies. **(D)** Exceptional point ($R_{\rm in}/R_{\rm out} = 0.4983$) where both real and imaginary frequencies coalesce. **(E)** Weak coupling regime ($R_{\rm in}/R_{\rm out} = 0.50$) characterized by the resonance crossing in the real frequencies, whereas avoided crossing in the imaginary frequencies. The colorful panels represent corresponding structural configurations in **(A)** and **(B)**.

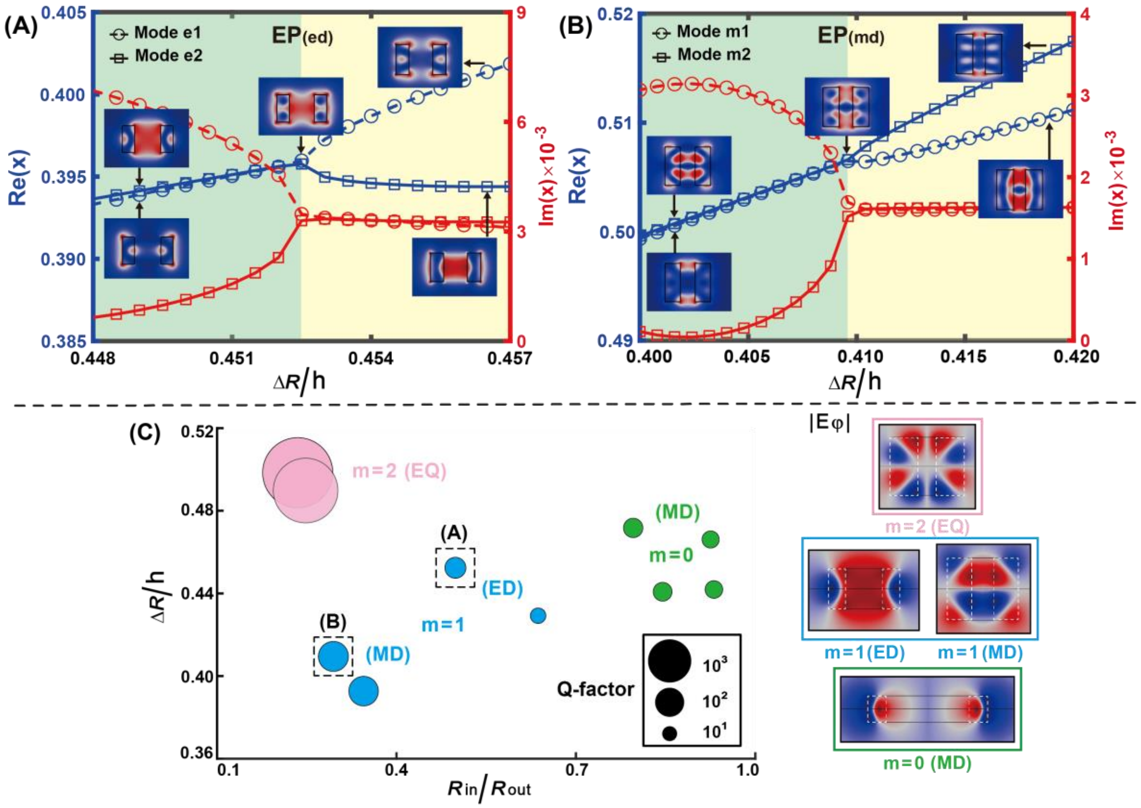

Fig. S8. Maps of multifaced EPs with different azimuthal indices and multipoles. (A) Simulated complex size parameter x of two eigenmodes as the functions of varying $\Delta R/h$ and radius ratio R_{in}/R_{out}. Magnetic dipole type EPs can be predicted in the position where both real and imaginary size parameters merge. The structural parameters of the chosen magnetic dipole type EP are: $\Delta R/h$ = 0.4095 and R_{in}/R_{out} = 0.2945. **(B)** The corresponding electric fields distribution of the eigenmodes with the same number of **(A)**. **(C)** Maps of EPs with different azimuthal indices and multipoles with the dependency of $\Delta R/h$ and R_{in}/R_{out}. Size of the dots represents the quality factor of EPs. The right panels show the distribution of azimuthal-component of the electric field.

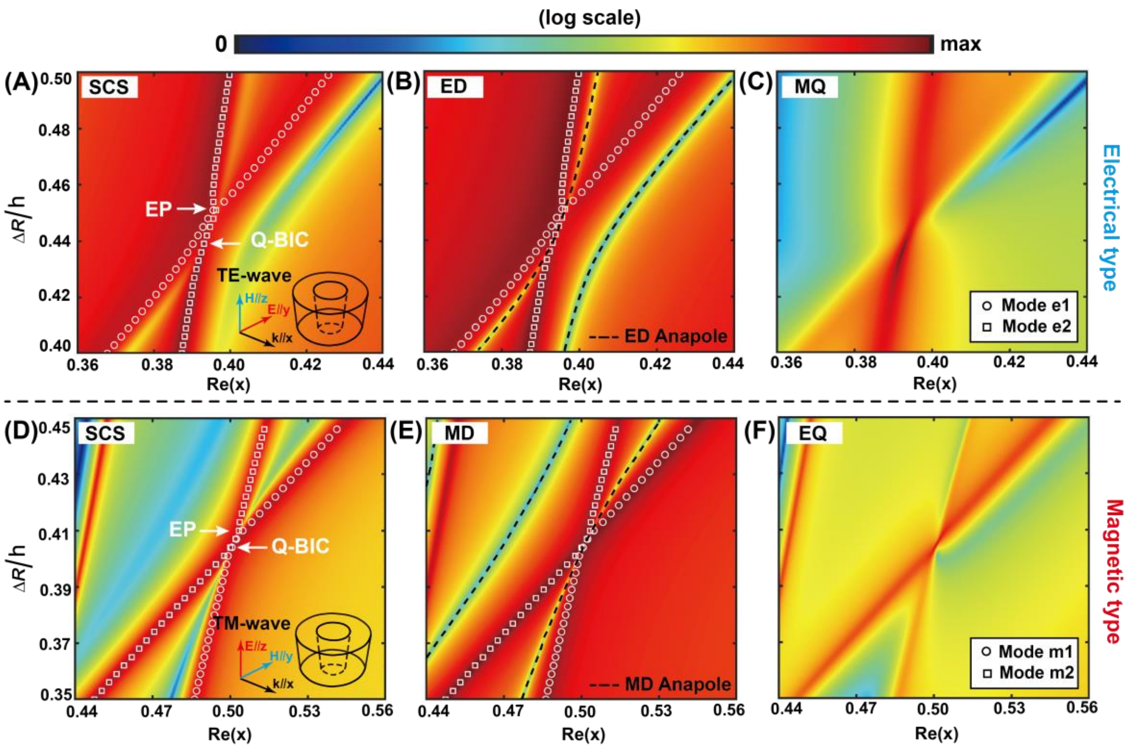

Fig. S9. Maps of the scattering cross sections with multipole analysis at electric and magnetic dipole contributed EPs. (A-C) are excited by the transverse electric (TE) wave to observe the electric type EPs and Q-BICs. **(D-F)** are excited by the transverse magnetic (TM) wave to observe the magnetic type EPs and Q-BICs. All maps show the dependency of aspect ratio $\Delta R/h$ and size parameter $x = \omega\Delta R/c = 2\pi f\Delta R/c$. The various types of the white dots indicate the real part of the eigenfrequencies in simulation. The white arrows indicate the positions of electric and magnetic type EPs and Q-BICs. The black dashed lines indicate the **(B)** ED anapole states and the **(E)** MD anapole states.

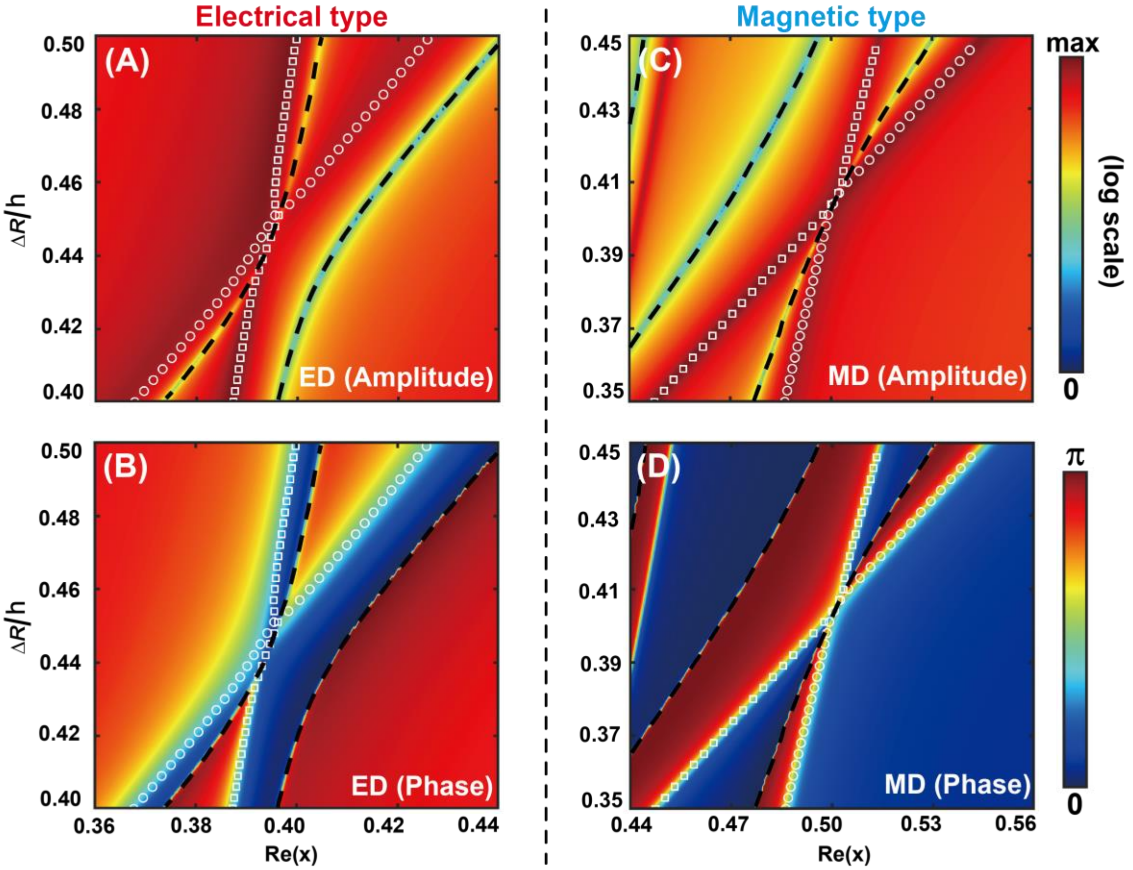

Fig. S10. Maps of the amplitude and phase properties in dipole contribution. (A) The amplitude of ED excited by the TE wave, the black dashed lines show the scattering dip in ED contribution. **(B)** The phase of ED excited by the TE wave, the black dashed lines show the π difference in the phase. The black dashed lines meet the condition of ED anapole states, i.e. The amplitude is the same but out-of-phase. **(C)** and **(D)** are the same with **(A)** and **(B)**, but MD anapole states excited by the TM wave.

Supplementary Tables

Table. S1. The values of R_{in}/R_{out} at different $\Delta R/h$ of electric EP in Fig. S8A

$\Delta R/h$	R_{in}/R_{out}
0.448	0.521
0.450	0.511
0.452	0.501
0.454	0.491
0.456	0.481
0.457	0.476

Table. S2. The values of R_{in}/R_{out} at different $\Delta R/h$ of magnetic EP in Fig. S8B

$\Delta R/h$	R_{in}/R_{out}
0.400	0.315
0.404	0.306
0.408	0.298
0.412	0.289
0.416	0.281
0.420	0.272

REFERENCES AND NOTES

1. K. Koshelev, Y. Kivshar, Dielectric resonant metaphotonics. *ACS Photonics* **8**, 102–112 (2021).

2. Y. Kivshar, The rise of Mie-tronics. *Nano Lett.* **22**, 3513–3515 (2022).

3. A. B. Evlyukhin, C. Reinhardt, A. Seidel, B. S. Luk'yanchuk, B. N. Chichkov, Optical response features of Si-nanoparticle arrays. *Phys. Rev. B* **82**, 045404 (2010).

4. A. García-Etxarri, R. Gómez-Medina, L. S. Froufe-Pérez, C. López, L. Chantada, F. Scheffold, J. Aizpurua, M. Nieto-Vesperinas, J. J. Sáenz, Strong magnetic response of submicron silicon particles in the infrared. *Opt. Express* **19**, 4815–4826 (2011).

5. A. I. Kuznetsov, A. E. Miroshnichenko, M. L. Brongersma, Y. S. Kivshar, B. Luk'yanchuk, Optically resonant dielectric nanostructures. *Science* **354**, aag2472 (2016).

6. C. Wang, Z. Fu, W. Mao, J. Qie, A. D. Stone, L. Yang, Non-Hermitian optics and photonics: From classical to quantum. *Adv. Opt. Photon.* **15**, 442 (2023).

7. M. V. Rybin, K. L. Koshelev, Z. F. Sadrieva, K. B. Samusev, A. A. Bogdanov, M. F. Limonov, Y. S. Kivshar, High-Q supercavity modes in subwavelength dielectric resonators. *Phys. Rev. Lett.* **119**, 243901 (2017).

8. A. A. Bogdanov, K. L. Koshelev, P. V. Kapitanova, M. V. Rybin, S. A. Gladyshev, Z. F. Sadrieva, K. B. Samusev, Y. S. Kivshar, M. F. Limonov, Bound states in the continuum and Fano resonances in the strong mode coupling regime. *Adv. Photonics* **1**, 016001 (2019).

9. N. Solodovchenko, K. Samusev, D. Bochek, M. Limonov, Bound states in the continuum in strong-coupling and weak-coupling regimes under the cylinder–ring transition. *Nanophotonics* **10**, 4347–4355 (2021).

10. S. Person, M. Jain, Z. Lapin, J. J. Sáenz, G. Wicks, L. Novotny, Demonstration of zero optical backscattering from single nanoparticles. *Nano Lett.* **13**, 1806–1809 (2013).

11. W. Liu, Y. S. Kivshar, Generalized Kerker effects in nanophotonics and meta-optics. *Opt. Express* **26**, 13085–13105 (2018).

12. A. E. Miroshnichenko, A. B. Evlyukhin, Y. F. Yu, R. M. Bakker, A. Chipouline, A. I. Kuznetsov, B. Luk'yanchuk, B. N. Chichkov, Y. S. Kivshar, Nonradiating anapole modes in dielectric nanoparticles. *Nat. Commun.* **6**, 8069 (2015).

13. H. Barati Sedeh, N. M. Litchinitser, From non-scattering to super-scattering with Mie-tronics. *Photonics Res.* **12**, 608–624 (2024).

14. A. Canós Valero, H. K. Shamkhi, A. S. Kupriianov, T. Weiss, A. A. Pavlov, D. Redka, V. Bobrovs, Y. Kivshar, A. S. Shalin, Superscattering emerging from the physics of bound states in the continuum. *Nat. Commun.* **14**, 4689 (2023).

15. Y. Ashida, Z. Gong, M. Ueda, Non-Hermitian physics. *Adv. Phys.* **69**, 249–435 (2020).

16. M.-A. Miri, A. Alù, Exceptional points in optics and photonics. *Science* **363**, eaar7709 (2019).

17..Kz demir, S. Rotter, F. Nori, L. Yang, Parity–time symmetry and exceptional points in photonics. *Nat. Mater.* **18**, 783–798 (2019).

18. H.-Z. Chen, T. Liu, H.-Y. Luan, R.-J. Liu, X.-Y. Wang, X.-F. Zhu, Y.-B. Li, Z.-M. Gu, S.-J. Liang, H. Gao, L. Lu, L. Ge, S. Zhang, J. Zhu, R.-M. Ma, Revealing the missing dimension at an exceptional point. *Nat. Phys.* **16**, 571–578 (2020).

19. A. Li, H. Wei, M. Cotrufo, W. Chen, S. Mann, X. Ni, B. Xu, J. Chen, J. Wang, S. Fan, C.-W. Qiu, A. Alù, L. Chen, Exceptional points and non-Hermitian photonics at the nanoscale. *Nat. Nanotechnol.* **18**, 706–720 (2023).

20. M. V. Berry, M. Wilkinson, Diabolical points in the spectra of triangles. *Proc. R. Soc. London Ser. A* **392**, 15–43 (1984).

21. J. Doppler, A. A. Mailybaev, J. Böhm, U. Kuhl, A. Girschik, F. Libisch, T. J. Milburn, P. Rabl, N. Moiseyev, S. Rotter, Dynamically encircling an exceptional point for asymmetric mode switching. *Nature* **537**, 76–79 (2016).

22. X.-L. Zhang, T. Jiang, C. T. Chan, Dynamically encircling an exceptional point in anti-parity-time symmetric systems: Asymmetric mode switching for symmetry-broken modes. *Light Sci. Appl. Ther.* **8**, 88 (2019).

23. A. Schumer, Y. Liu, J. Leshin, L. Ding, Y. Alahmadi, A. Hassan, H. Nasari, S. Rotter, D. Christodoulides, P. LiKamWa, M. Khajavikhan, Topological modes in a laser cavity through excep-tional state transfer. *Science* **375**, 884–888 (2022).

24. Z. Lin, H. Ramezani, T. Eichelkraut, T. Kottos, H. Cao, D. N. Christodoulides, Unidirectional invisibility induced by PT-symmetric periodic structures. *Phys. Rev. Lett.* **106**, 213901 (2011).

25. L. Feng, Y.-L. Xu, W. S. Fegadolli, M.-H. Lu, J. E. B. Oliveira, V. R. Almeida, Y.-F. Chen, A. Scherer, Experimental demonstration of a unidirectional reflectionless parity-time meta-material at optical frequencies. *Nat. Mater.* **12**, 108–113 (2013).

26. Y. Xu, L. Li, H. Jeong, S. Kim, I. Kim, J. Rho, Y. Liu, Subwavelength control of light transport at the exceptional point by non-Hermitian metagratings. *Sci. Adv.* **9**, eadf3510 (2023).

27. Y. Choi, C. Hahn, J. W. Yoon, S. H. Song, P. Berini, Extremely broadband, on-chip optical nonreciprocity enabled by mimicking nonlinear anti-adiabatic quantum jumps near exceptional points. *Nat. Commun.* **8**, 14154 (2017).

28. Y. Sun, W. Tan, H.-q. Li, J. Li, H. Chen, Experimental demonstration of a coherent perfect absorber with PT phase transition. *Phys. Rev. Lett.* **112**, 143903 (2014).

29. C. Wang, X. Jiang, G. Zhao, M. Zhang, C. W. Hsu, B. Peng, A. D. Stone, L. Jiang, L. Yang, Electromagnetically induced transparency at a chiral exceptional point. *Nat. Phys.* **16**, 334–340 (2020).

30. J. Yu, B. Ma, A. Ouyang, P. Ghosh, H. Luo, A. Pattanayak, S. Kaur, M. Qiu, P. Belov, Q. Li, Dielectric super-absorbing metasurfaces via PT symmetry breaking. *Optica* **8**, 1290–1295 (2021).

31. W. Chen, Ş. Kaya Özdemir, G. Zhao, J. Wiersig, L. Yang, Exceptional points enhance sensing in an optical microcavity. *Nature* **548**, 192–196 (2017).

32. W. Mao, Z. Fu, Y. Li, F. Li, L. Yang, Exceptional–point–enhanced phase sensing. *Sci. Adv.* **10**, eadl5037 (2024).

33. W. Tang, X. Jiang, K. Ding, Y.-X. Xiao, Z.-Q. Zhang, C. T. Chan, G. Ma, Exceptional nexus with a hybrid topological invariant. *Science* **370**, 1077–1080 (2020).

34. N. Wu, K. Cui, Q. Xu, X. Feng, F. Liu, W. Zhang, Y. Huang, On-chip mechanical exceptional points based on an optomechanical zipper cavity. *Sci. Adv.* **9**, eabp8892 (2023).

35. K. Bai, L. Fang, T.-R. Liu, J.-Z. Li, D. Wan, M. Xiao, Nonlinearity-enabled higher-order exceptional singularities with ultra-enhanced signal-to-noise ratio. *Natl. Sci. Rev.* **10**, nwac259 (2023).

36. T. Gao, E. Estrecho, K. Y. Bliokh, T. C. H. Liew, M. D. Fraser, S. Brodbeck, M. Kamp, C. Schneider, S. Höfling, Y. Yamamoto, F. Nori, Y. S. Kivshar, A. G. Truscott, R. G. Dall, E. A. Ostrovskaya, Observation of non-Hermitian degeneracies in a chaoticexciton-polariton billiard. *Nature* **526**, 554–558 (2015).

37. M. A. Masharin, T. Oskolkova, F. Isik, H. Volkan Demir, A. K. Samusev, S. V. Makarov, Giant ultrafast all-optical modulation based on exceptional points in exciton–polariton per-ovskite metasurfaces. *ACS Nano* **18**, 3447–3455 (2024).

38. F. Ding, Y. Deng, C. Meng, P. C. V. Thrane, S. I. Bozhevolnyi, Electrically tunable topological phase transition in non-Hermitian optical MEMS metasurfaces. *Sci. Adv.* **10**, eadl4661 (2024).

39. B. Zhen, C. W. Hsu, Y. Igarashi, L. Lu, I. Kaminer, A. Pick, S.-L. Chua, J. D. Joannopoulos, M. Soljačić, Spawning rings of exceptional points out of Dirac cones. *Nature* **525**, 354–358 (2015).

40. H. Zhou, C. Peng, Y. Yoon, C. W. Hsu, K. A. Nelson, L. Fu, J. D. Joannopoulos, M. Soljačić, B. Zhen, Observation of bulk Fermi arc and polarization half charge from paired exceptional points. *Science* **359**, 1009–1012 (2018).

41. M. Lawrence, N. Xu, X. Zhang, L. Cong, J. Han, W. Zhang, S. Zhang, Manifestation of *PT* symmetry breaking in polarization space with terahertz metasurfaces. *Phys. Rev. Lett.* **113**, 093901 (2014).

42. Z. Yang, P.-S. Huang, Y.-T. Lin, H. Qin, J. Zúñiga-Pérez, Y. Shi, Z. Wang, X. Cheng, M.-C. Tang, S. Han, B. Kanté, B. Li, P. C. Wu, P. Genevet, Q. Song, Creating pairs of exceptional points for arbitrary polarization control: Asymmetric vectorial wavefront modulation. *Nat. Commun.* **15**, 232 (2024).

43. T. S. Bai, W. Z. Wang, X. Zhang, T. J. Cui, Exceptional point in a microwave plasmonic dipole resonator for sub-microliter solution sensing. *Adv. Funct. Mater.* **34**, 2312170 (2024).

44. Z. Liao, X. Peng, L. Liu, Y. Xu, K. Xu, B. Pan, G. Q. Luo, Y. Liu, Microwave plasmonic exceptional points for enhanced sensing. *Laser Photonics Rev.* **17**, 2300276 (2023).

45. Q. Song, M. Odeh, J. Zúñiga-Pérez, B. Kanté, P. Genevet, Plasmonic topological metasurface by encircling an exceptional point. *Science* **373**, 1133–1137 (2021).

46. A. Guo, G. J. Salamo, D. Duchesne, R. Morandotti, M. Volatier-Ravat, V. Aimez, G. A. Siviloglou, D. N. Christodoulides, Observation of *PT*-symmetry breaking in complex optical potentials. *Phys. Rev. Lett.* **103**, 093902 (2009).

47. J.-H. Park, A. Ndao, W. Cai, L. Hsu, A. Kodigala, T. Lepetit, Y.-H. Lo, B. Kanté, Symmetry-breaking-induced plasmonic exceptional points and nanoscale sensing. *Nat. Phys.* **16**, 462–468 (2020).

48. A. Canós Valero, V. Bobrovs, T. Weiss, L. Gao, A. S. Shalin, Y. Kivshar, Bianisotropic exceptional points in an isolated dielectric nanoparticle. *Phys. Rev. Res.* **6**, 013053 (2024).

49. N. S. Solodovchenko, K. B. Samusev, M. F. Limonov, Quadruplets of exceptional points and bound states in the continuum in dielectric rings. *Phys. Rev. B* **109**, 075131 (2024).

50. P. Lalanne, W. Yan, K. Vynck, C. Sauvan, J.-P. Hugonin, Light interaction with photonic and plasmonic resonances. *Laser Photonics Rev.* **12**, 1700113 (2018).

51. S. N. Sheikholeslami, A. García-Etxarri, J. A. Dionne, Controlling the interplay of electric and magnetic modes via Fano-like plasmon resonances. *Nano Lett.* **11**, 3927–3934 (2011).

52. S. Gladyshev, K. Frizyuk, A. Bogdanov, Symmetry analysis and multipole classification of eigenmodes in electromagnetic resonators for engineering their optical properties. *Phys. Rev. B* **102**, 075103 (2020).

53. A. C. Valero, Z. Sztranyovszky, E. A. Muljarov, A. Bogdanov, T. Weiss, Exceptional bound states in the continuum. arXiv: https://arxiv.org/abs/2309.01501 (2023).

54. S. Gladyshev, O. Pashina, A. Proskurin, A. Nikolaeva, Z. Sadrieva, M. Petrov, A. Bogdanov, K. Frizyuk, Fast Simulation of Light Scattering and Harmonic Generation in Axially Symmetric Structures in COMSOL. *ACS Photonics* **11**, 404–418 (2024).

55. C. Larsson, M. Gustafsson, Wideband Measurements of the Forward RCS and the Extinction Cross Section. *ACES Journal* **28**, 1145–1152 (2013).

56. M. Odit, K. Koshelev, S. Gladyshev, K. Ladutenko, Y. Kivshar, A. Bogdanov, Observation of Supercavity Modes in Subwavelength Dielectric Resonators. *Adv. Mater.* **33**, 2003804 (2021).

57. W. Suh, Z. Wang, S. Fan, Temporal coupled-mode theory and the presence of non-orthogonal modes in lossless multimode cavities. *IEEE J. Quantum Electron.* **40**, 1511–1518 (2004).

58. Z. Ruan, S. Fan, Temporal coupled-mode theory for Fano resonance in light scattering by a single obstacle. *J. Phys. Chem. C* **114**, 7324–7329 (2010).

59. R. Alaee, C. Rockstuhl, I. Fernandez-Corbaton, An electromagnetic multipole expansion beyond the long-wavelength approximation. *Optics Commun.* **407**, 17–21 (2018).

60. E. A. Gurvitz, K. S. Ladutenko, P. A. Dergachev, A. B. Evlyukhin, A. E. Miroshnichenko, A. S. Shalin, The high-order toroidal moments and anapole states in all-dielectric photonics. *Laser Photonics Rev.* **13**, 1800266 (2019).

61. C. Sauvan, J. P. Hugonin, I. S. Maksymov, P. Lalanne, Theory of the spontaneous optical emission of nanosize photonic and plasmon resonators. *Phys. Rev. Lett.* **110**, 237401 (2013).

62. Q. Bai, M. Perrin, C. Sauvan, J.-P. Hugonin, P. Lalanne, Efficient and intuitive method for the analysis of light scattering by a resonant nanostructure. *Opt. Express* **21**, 27371–27382 (2013).

63. W. Yan, R. Faggiani, P. Lalanne, Rigorous modal analysis of plasmonic nanoresonators. *Phys. Rev. B* **97**, 205422 (2018).

64. J. D. Jackson, *Classical Electrodynamics* (John Wiley, ed. 3, 1999).

65. U. Fano, Effects of configuration interaction on intensities and phase shifts. *Phys. Rev.* **124**, 1866 (1961).

66. M. F. Limonov, M. V. Rybin, A. N. Poddubny, Y. S. Kivshar, Fano resonances in photonics. *Nat. Photonics* **11**, 543–554 (2017).

67. M. F. Limonov, Fano resonance for applications. *Adv. Opt. Photonics* **13**, 703–771 (2021).
